# Mechanistic insights into protein-mediated Zinquin fluorescence

**DOI:** 10.5802/crchim.482

**Published:** 2026-11-04

**Authors:** Natalie B. Schulte, Addison M. Duda, Sara M. Tuck, Kelly N. Chacón, Benjamin G. Bobay, Michael J. Therien, Andrew M. Dale, Katherine J. Franz

**Affiliations:** a Department of Chemistry, Duke University, 124 Science Drive, Durham, NC 27710, USA; b Department of Chemistry, Reed College, Portland, OR 97202, USA

**Keywords:** Fluorescent probes, Zinc metalloproteins, Protein–probe interactions, Zinc biology, Metal-responsive fluorophores

## Abstract

Zinquin (ZQ) is a small-molecule fluorescent probe used to monitor labile zinc in biological systems based on green fluorescence emission arising from the formation of [Zn(ZQ)_2_]^2−^ complexes. However, studies in cells and protein-rich environments frequently report a pronounced blue-shifted emission, suggesting alternative interaction modes that complicate the interpretation of zinc imaging experiments. The nature of the interactions driving the blue shift is not fully understood. Here, we use the metallo-β-lactamase NDM-1 as a chemically and structurally well-defined model to elucidate the molecular origins of ZQ fluorescence in a zinc-binding protein. The NDM-1 has two distinct Zn^2+^ ions in its active site, enabling direct assessment of zinc speciation, enzymatic activity, and structural integrity. Through an integrated approach combining fluorescence spectroscopy, enzyme activity assays, inductively coupled plasma mass spectrometry, X-ray absorption spectroscopy, and nuclear magnetic resonance, we identify two sources of ZQ fluorescence for this protein: formation of [Zn(ZQ)_2_]^2−^ by stripping the weaker bound zinc, and a zinc-mediated ternary complex formed by NDM-1, Zn^2+^, and ZQ. This 1:1:1 complex localizes at the higher affinity zinc-binding site, where ZQ interacts concurrently with protein-bound zinc and specific residues of the NDM-1 active site. These nonpolar interactions generate a blue-shifted emission that is spectroscopically distinct from free zinc–ZQ complexes. This study characterizes the ternary complex that drives a protein-mediated fluorescence mechanism for ZQ and highlights the need to consider probe–protein interactions when interpreting fluorescent signals in chemical biology studies.

## 1. Introduction

Metal ions play important roles in complex biological systems, necessitating sensitive and reliable methods to interrogate their distribution and function. Metal-responsive fluorescent probes have become widely used tools for investigating the spatial and temporal changes of essential metal ions within cells and tissues under diverse conditions [1–5]. Accurate interpretation of these fluorescence responses critically depends on an understanding of the physical and chemical properties underlying metal recognition, including the speciation of metal pools that are accessible to the probe [6].

Zinquin (ZQ) was among the first fluorescent probes developed to detect intracellular zinc (Zn^2+^) in live cells [7,8]. Fluorescent turn-on of ZQ occurs upon complex formation of [Zn(ZQ)_2_]^2−^, yielding fluorescence emission with *λ*_max_ ~ 500 nm at biological pH with a conditional affinity of log*K*_a_ = 13.5 [9]. The ZQ probe was introduced as an alternative to the fluorescent dye 6-methoxy-8-*p*-toluene sulfonamide quinoline (TSQ), which also forms a 2:1 stoichiometric complex with Zn^2+^ but lacks membrane permeability [6]. Cellular uptake of ZQ is facilitated by its ethyl ester derivative (ZQ_EE_), which undergoes intracellular hydrolysis to yield the negatively charged acid form (ZQ_A_; Figure 1) although esterification is not strictly required for membrane permeability [10].

Early studies assumed that fluorescent probes such as TSQ and ZQ_A_ report on intracellular Zn^2+^ by engaging labile metal pools that are accessible to ligand-exchange reactions, resulting in the formation of discrete fluorescent Zn–probe complexes [11]. However, experiments in cells, isolated proteins, and peptides have frequently revealed a Zn^2+^-dependent fluorescence response from ZQ_A_ that is blue-shifted relative to the expected emission maximum of the [Zn(ZQ_A_)_2_]^2−^ complex (490–500 nm) depending on buffer conditions [12–14]. Studies from the Petering lab have provided compelling evidence for a model wherein the blue-shifted fluorescence arises from ZQ_A_ interacting with “proteome Zn^2+^” [12,15,16]. In this model, emission at 492 nm reports on labile Zn^2+^ that is sequestered as [Zn(ZQ_A_)_2_]^2−^, while blue-shifted emission reports on Zn^2+^ that remains protein-bound upon interaction with ZQ_A_. Proteins with fully saturated Zn^2+^ coordination sites would be inert to ZQ_A_, while those possessing open coordination sites could engage ZQ_A_ to form Zn-mediated ternary complexes. Supporting this model, studies using small-molecule ligands containing nitrogen and oxygen donor atoms have demonstrated the propensity of ZQ_A_ to form Zn-mediated ternary complexes [17]. Although these systems exhibit a range of quantum yields, they generally do not display pronounced shifts in emission maxima. As a result, despite indirect evidence for ternary complex formation, the lack of direct biophysical characterization of a ZQ_A_–Zn–protein complex has left unresolved questions about the origin of blue-shifted emission.

To address these unresolved questions, we used the well-characterized di-Zn metallo-β-lactamase NDM-1 as a model system to investigate interactions with ZQ_A_ that drive the blue fluorescence shift. We purified the mature, translocated version of NDM-1 from *Escherichia coli*, which lacks its physiological N-terminal signal sequence that is cleaved following periplasmic translocation. The NDM-1 binds 2 molar equivalents of Zn^2+^ (holo-NDM-1) in its solvent-exposed active site, enabling direct correlation between Zn^2+^ coordination and enzymatic activity [18]. The two Zn^2+^-binding sites differ markedly in affinity, with the higher affinity site (H^120^, H^122^, H^189^) having a *K*_D_ between that of common Zn^2+^ chelators PAR and EDTA and the lower affinity site (D^124^, C^208^, H^250^) having a *K*_D_ ~ 7.4 μM [19,20]. Because NDM-1 has well-characterized Zn^2+^ coordination sites and previous NDM-1 inhibitors have been proposed to form Zn^2+^ - mediated ternary complexes [20,21], it provides an ideal platform for dissecting the chemical basis of ZQ_A_ fluorescence responses in Zn^2+^-containing proteins. Utilizing fluorescence spectroscopy, enzyme activity assays, inductively coupled plasma mass spectrometry (ICP-MS), high-performance liquid chromatography/mass spectrometry (HPLC/MS), X-ray absorption spectroscopy (XAS), and nuclear magnetic resonance (NMR), we have characterized two pathways of Zn^2+^-dependent ZQ fluorescence: one arising from the sequestration of the weakly bound Zn^2+^ as a [Zn(ZQ_A_)_2_]^2−^ complex and a second blue-shifted response mediated by protein-bound Zn^2+^, situating ZQ_A_ in a hydrophobic pocket with specific protein interactions that shift both the intensity and *λ*_max_ of its fluorescence emission.

## 2. Results

### 2.1. Fluorescence spectra with blue shifts are unique to the presence of NDM-1, Zn^2+^, and ZQ_A_

Titration of ZQ_A_ into holo-NDM-1 initially produced a steady increase in fluorescence intensity with a maximum at 500 nm, consistent with the formation of the [Zn(ZQ_A_)_2_]^2+^ complex at pH 7.5 (Figure 2A,B). Beyond 2 equivalents of ZQ_A_, *λ*_max_ shifted to 488 nm, with a continued increase in intensity up to 7 molar equivalents of ZQ_A_. Fluorescence was not observed in the absence of Zn^2+^ (Figure 2C), verifying that ZQ_A_ does not form an emissive species with the apoprotein. These patterns parallel prior observations that ZQ_A_ fluorescence is both blue-shifted and more intense for protein-containing samples compared to [Zn(ZQ_A_)_2_]^2−^ alone [15]. Our fluorescence data suggest there are two predominant emissive species giving rise to the spectra in Figure 2A: the isolated [Zn(ZQ_A_)_2_]^2+^ complex and another that contains NDM-1, Zn^2+^, and ZQ_A_. The decrease in fluorescence intensity observed at concentrations above 7 molar equivalents of added ZQ_A_ indicates loss or other distortion of this tripartite complex (Figures 2A,B and S2).

To understand the electronic basis of the blue shift seen when NDM-1, Zn^2+^, and ZQ_A_ are present in solution, the fluorescence of the [Zn(ZQ_A_)_2_]^2−^ complex was tested in solvents spanning a range of dielectric constants (Figure 2D). The complex exhibited an emission maximum of 500 nm in water (dielectric constant ~80) [22] and buffer, shifted slightly to 498 nm in acetonitrile (dielectric constant ~37) [22], and more substantially to 492 nm in tetrahydrofuran (THF), a low-dielectric solvent (7–8) [23] comparable to the interior of a protein [24]. This THF-induced blue shift parallels the shift seen during ZQ_A_ titration into holo-NDM-1. Fluorescence intensity also varied depending on solvent conditions, with Storage Buffer producing the largest enhancement, followed by the organic solvents. An intensity increase was also observed in the presence of the protein (Figure 2A,B). Together, these solvent studies support the conclusion that the blue shift arises from interactions that position a Zn-bound ZQ_A_ unit within a hydrophobic, protein-like environment.

### 2.2. ZQ_A_ inhibits NDM-1 enzymatic activity

Previous literature has reported that Zn^2+^-binding compounds can inhibit the activity of NDM-1, either from disrupting access to the catalytic site or by removal of the active-site Zn^2+^ ions. To determine the effect of ZQ_A_ on the activity of NDM-1, enzyme inhibition experiments were performed on NDM-1 to quantify IC_50_ values for ZQ_A_. The chromogenic β-lactam nitrocefin was used to measure relative enzyme activity after incubation with ZQ_A_. Pyrithione, a known Zn^2+^ chelator and competitive inhibitor that forms a ternary complex with holo-NDM-1, was used as a positive control and exhibited an IC_50_ value consistent with reported values (133 ± 8 μM) [25]. The ZQ_A_ inhibited NDM-1 with an IC_50_ of 2.39 ± 0.09 μM (Figure 3), indicating perturbation of the active site through Zn^2+^ removal, ternary complex formation, or both.

### 2.3. Pseudo-titration of ZQ_A_ into holo-NDM-1 fraction supports ternary complex formation

The parallel observations of ZQ_A_’s fluorescence shift and enzyme inhibitory activity indicate biochemical disruption of holo-NDM-1 by ZQ_A_. To investigate how ZQ_A_ interacts with holo-NDM-1, we quantified the ratio of ZQ_A_ to Zn^2+^ to NDM-1 following treatment of holo-NDM-1 with increasing concentrations of ZQ_A_ and using spin filtration to fractionate high-molecular-weight species that contain protein complexed with Zn^2+^ and/or ZQ_A_ (retained samples) from low-molecular-weight species of ZQ_A_ or [Zn(ZQ_A_)_2_]^2−^ not interacting with protein (eluted samples) (Figure S1). Bradford assays confirmed that all protein remained in the high-molecular-weight, retained portion of the samples. All fractions were analyzed via fluorescence, UV–vis, and ICP-MS, and the retained fractions were analyzed by HPLC/MS.

Fluorescence of the eluted and retained samples varied significantly, with eluted samples having an observed *λ*_max_ of 500 nm and the retained samples having a blue fluorescence shift with *λ*_max_ of 473 nm (Figure 4A,B). The retained and eluted samples also showed a large difference in intensity, partly attributable to dilution of the eluted samples due to washing steps. This fluorescence shift indicates the presence of NDM-1, Zn^2+^, and ZQ_A_ in the retained portion. The UV–vis absorption spectra of the eluted and retained samples also differed from one another and were compared to a control titration of Zn^2+^ into ZQ_A_ for a qualitative assessment of the species present in fractionated samples. The ZQ_A_ has unique features at 240, 273, and 336 nm that shift to 264 and 360 nm upon Zn^2+^ titration (Figure 4C). The spectra in Figure 4D for retained samples show a strong peak at 268 nm that differs from holo-NDM-1 alone and aligns with the features of ZQ_A_. Broad, weak absorbances centered at ~360 nm (Figure 4D) further suggest the presence in the retained samples of ZQ_A_, which absorbs in this region with variations induced by Zn^2+^ (Figure 4C). Eluted samples contained features at 264, 336, and ~360 nm that increased with the amount of ZQ_A_ in the samples prior to fractionation (Figure 4E). The broad absorbance centered at 360 nm is perhaps the most distinctive feature indicating the presence of the [Zn(ZQ_A_)_2_]^2−^ complex, while the features at 264 nm and 336 nm suggest a mixture of free ZQ_A_ and [Zn(ZQ_A_)_2_]^2−^ complex across the range of eluted samples.

Quantitative determination of Zn^2+^ content in the retained samples by ICP-MS revealed a ZQ_A_-dependent decrease in Zn^2+^ associated with NDM-1. Untreated holo-NDM-1 contained 2.7 ± 0.1 molar equivalents of Zn^2+^. Incubation with 5, 10, or 15 molar equivalents of ZQ_A_ reduced Zn^2+^ retention to 1.5 ± 0.1 equivalents, while 20 molar equivalents and more further reduced Zn^2+^ to 1 per protein (Figure 4F). The HPLC/MS analysis of matched samples showed that ~1 molar equivalent of ZQ_A_ remained in the retained samples treated with 5–20 molar equivalents of ZQ_A_, with a second equivalent detected only at substantially higher ZQ_A_ levels (Figure 4F; see Figures S4–S6 for standard curves and quality-control experiments). Together with the fluorescence and absorbance data, these results support the formation of a discrete 1:1:1 ZQ_A_–Zn–NDM-1 complex when holo-NDM-1 is treated with ZQ_A_.

### 2.4. EXAFS and ^15^N–^1^H HSQC NMR support a Zn^2+^-mediated ternary complex

To determine how the coordination environment surrounding Zn^2+^ changes in the presence of NDM-1 and ZQ_A_, we used extended X-ray absorption fine structure (EXAFS) spectroscopy. When apo-NDM-1 is treated with 0.9 molar equivalents of Zn^2+^, the data are best fit by a model that has Zn^2+^ bound by four multiscattering N atoms of His residues at an average distance of ~2.01 Å (Figure S7A, Table S1). This coordination is consistent with Zn coordinated at the high-affinity site in NDM-1, with slight variation from reported crystal structures due to increased active-site flexibility when only one Zn^2+^ is present [26]. In contrast, Zn complexed with ZQ_A_ alone exhibited a mixed primary coordination sphere composed of four N/O donors distributed over two distinct Zn^2+^–donor distances (~1.98 and ~2.10 Å), with additional C, O/N, and S scatterers in the outer coordination shell, characteristic of the [Zn(ZQ_A_)_2_]^2−^ complex (Figure S7B, Table S1). When holo-NDM-1 was treated with 5 molar equivalents of ZQ_A_ and washed via spin filtration to remove unbound components, the resulting EXAFS spectrum was best fit by a composite coordination environment. The primary sphere included one multiscattering N atom at 1.19 Å along with N/O donors at distances over-lapping those observed for both protein- and ZQ_A_-bound Zn. The outer coordination shell contained contributions consistent with both protein-derived and ligand-derived O/N and S scatterers (Figure S7C, Table S1). An overlay of the Fourier-transformed spectra reveals clear spectral features shared with both mono-Zn NDM-1 and ZQ_A_–Zn controls, indicating partial retention of native protein coordination coupled with direct ligand involvement (Figure 5). Together, these fits support the formation of a Zn^2+^ center jointly coordinated by NDM-1 and ZQ_A_, consistent with a Zn^2+^-mediated ternary complex.

To determine the binding location of ZQ_A_ within the protein, NDM-1 was isotopically labeled with ^15^N and subjected to ^15^N–^1^ H HSQC NMR spectroscopy. The HSQC cross-peaks for all residues of our purified holo ^15^N-NDM-1 match assignments reported in the literature [27,28], with the exception of the last four C-terminal residues. Our construct and purification protocol involves the removal of an N-terminal 6xHis tag to enable accurate characterization of the binding of Zn^2+^ and ZQ_A_ to the protein without potential interference of a non-native metal-binding region. In comparison, the reported spectra come from a protein construct containing an intact C-terminal 6xHis tag [27,28]. The unresolved cross-peaks in our spectra are likely due to this variation in protein constructs.

To confirm appropriate Zn^2+^ loading of ^15^N-NDM-1, a Zn^2+^ titration was performed with apo ^15^N-NDM-1 (Figure S18), which was prepared by dialyzing the recombinant protein against EDTA. The observed ^15^N–^1^H HSQC cross-peaks for apo ^15^N-NDM-1 are consistent with literature spectra [26,29]. Upon the addition of one equivalent of ZnSO_4_, significant perturbations in ^15^N–^1^H HSQC cross-peaks are observed, indicating that the structure of NDM-1 is altered from apo- to mono-Zn. The addition of a second equivalent of ZnSO_4_ results in the sharpening of the new ^15^N–^1^H HSQC cross-peaks, indicating that mono-Zn NDM-1 and holo-NDM-1 have similar structures, but holo-NDM-1 is more stable. These results are consistent with literature reports [26–28]. The addition of a third equivalent of ZnSO_4_ results in no spectral changes, which shows that holo-NDM-1 does not bind additional Zn^2+^, suggesting that any adventitiously adsorbed Zn^2+^ does not influence structure.

Large chemical shift perturbations from ZQ_A_ addition to holo ^15^N-NDM-1 were observed only after the addition of two equivalents of ZQ_A_ (Figure 6A). This observation is consistent with our parallel characterizations of ZQ_A_ stripping the first equivalent of Zn^2+^ to form [Zn(ZQ_A_)_2_]^2−^ and mono Zn NDM-1, with additional ZQ_A_ then interacting with mono-Zn NDM-1. The subsequent addition of ZQ_A_ up to four equivalents enhanced the intensity of the new, perturbed ^15^N–^1^H HSQC cross-peaks observed at two equivalents of ZQ_A_, indicating that the population of NDM-1 bound to ZQ_A_ had increased.

To confirm if the interaction of ZQ_A_ with NDM-1 is dependent on Zn^2+^, we performed an identical titration of ZQ_A_ against apo ^15^N-NDM-1 (Figure S19). No chemical shifts were observed for any cross-peaks, corroborating our parallel results (Figure 2C) and confirming that Zn^2+^ is required for ZQ_A_ to interact with NDM-1. To confirm that ZQ_A_ can bind to a mono-Zn NDM-1, we performed a ZQ_A_ titration against apo ^15^N-NDM-1 with one equivalent of ZnSO_4_. Chemical shift perturbations were observed upon the addition of ZQ_A_. Notably, significant chemical shift perturbations were observed upon addition of the first equivalent of ZQ_A_ to mono-Zn ^15^N-NDM-1. Since mono-Zn NDM-1 lacks the lower affinity Zn^2+^ , ZQ_A_ immediately binds to NDM-1 and perturbs its structure. Together, the ^15^N–^1^H HSQC NMR and EXAFS results support ZQ_A_ binding to mono-Zn NDM-1, after stripping away the first labile Zn^2+^ from holo-NDM-1, to provide a ternary complex.

To determine the binding pose of ZQ_A_ to NDM-1, we aggregated our characterization results to develop a HADDOCK model. HADDOCK is an algorithm that accommodates experimental data as constraints to steer molecular docking. Titrations of holo-NDM-1 characterized by ^15^N–^1^H HSQC NMR revealed 50 residue cross-peaks that disappeared on the addition of ZQ_A_ (see Supporting Data). Cross-peaks identified as having disappeared during the titration have the greatest environmental change promoted by the binding of a new molecule. Of the cross-peaks that disappeared, 72% were within 15 Å of the zinc ions and 38% were within 10 Å. The perturbation of residues near the active site of NDM-1 indicates that ZQ_A_ is binding in this location.

To define residues that are directly or indirectly involved with the association of ZQ_A_ with NDM-1, protein molecular dynamics (MD) were performed on mono-Zn NDM-1 with the Zn^2+^ ion localized to H^120^, H^122^, and H^189^, supported by our previous observations and literature reports. Residues with greater than 40% solvent accessibility across the converged MD simulation and within 15 Å of the Zn^2+^ were used to guide HADDOCK docking.

The ZQ_A_ dianion (ZQ_A_^2−^), deprotonated at its carboxylic acid and sulfonamide nitrogen, was used as the protonation state for HADDOCK docking. The ZQ_A_^2−^ is known to bind Zn^2+^ as a bidentate ligand through its two nitrogen donors (Figure 1). The HADDOCK modeling of ZQ_A_^2−^ to MD-optimized mono-Zn NDM-1 produced well-defined binding poses with strong clustering, indicating a preferred and stable binding mode (Figure 6B). A tetrahedral coordination site for Zn^2+^ is provided by N donors from H^120^ and H^122^ of NDM-1 at distances of 2.40 Å and 2.60 Å, respectively, and the quinoline and sulfonamide of ZQ_A_^2−^ at distances of 2.20 Å and 2.15 Å, respectively. The H^189^ presents a possible fifth ligand situated at a longer distance. Beyond metal coordination, the modeled complex reveals stabilizing aromatic interactions that position ZQ_A_^2−^ within the active site. Notably, π–π stacking interactions are observed between H^250^ and ZQ_A_’s *p*-tolyl group as well as H^122^ and ZQ_A_’s quinoline group. These intermolecular forces act as levers on either side of the fluorescent probe to distort the *p*-tolyl group (Figure 6C).

Alignment of the modeled ZQ_A_ ternary complex (ZQ_A_ molecule omitted for clarity) with those of reported structures of holo-NDM-1 (PDB: 3Q6X) and mono-Zn NDM-1 (PDB: 3SFP) reveals that ternary complex formation and changes in metalation state are accommodated primarily by side-chain rearrangements rather than global protein unfolding (Figure 6D,E). This conclusion is consistent with ^15^N–^1^H HSQC NMR titration data, which show localized chemical shift perturbations indicative of conformational flexibility without large-scale structural disruption.

## 3. Discussion and conclusions

In this work, we establish that the blue-shifted fluorescence and enhanced emission intensity of ZQ_A_ arise from the formation of a discrete ZQ_A_–Zn–NDM-1 ternary complex, rather than from simple Zn^2+^ chelation or nonspecific environmental effects. Although prior studies have proposed ternary complex formation to explain the fluorescence behavior of ZQ_A_ and TSQ probes, direct structural and biophysical validation has been lacking. Our data provide convergent evidence collected from a suite of different experimental methods that ZQ_A_ forms a defined 1:1:1 complex with Zn^2+^ bound in the active site of NDM-1.

Fluorescence titration experiments reveal that the characteristic blue shift and intensity increase occur only when NDM-1, Zn^2+^, and ZQ_A_ are simultaneously present and only after the weakly bound Zn^2+^ has been removed from the enzyme as [Zn(ZQ_A_)_2_]^2−^ (Figure 2). The observed fluorescence *λ*_em_ of 488 nm for the titration in Figure 2 is therefore a composite of both [Zn(ZQ_A_)_2_]^2−^ (*λ*_em_ = 500 nm) and ZQ_A_–Zn–NDM-1 (*λ*_em_ = 473 nm) present in solution as verified by the spectra of these isolated species after fractionation (Figure 4A). This behavior closely parallels a previous report for di-Zn enzyme alcohol dehydrogenase, where the redistribution of metal ions enables the formation of distinct fluorescent species [15]. Together, these observations argue for a model in which ZQ_A_ signals both labile and protein-bound Zn, even within the same protein.

Solvent dielectric studies further clarify the microenvironment experienced by ZQ_A_ in the protein ternary complex. The ZQ_A_–Zn fluorescence in low-dielectric solvents such as THF exhibits a blue shift and enhanced intensity comparable to that observed upon binding to NDM-1, whereas more polar solvents fail to reproduce this behavior (Figure 2). Dielectric constants refer to the polarity of a solvent, and proteins can display a variety of dielectric constants for different regions. Protein surfaces are generally solvent-exposed with relatively high dielectric constants ~20–30, whereas buried hydrophobic regions have dielectric constants below 10 [24]. The active site of NDM-1, aside from the Zn^2+^ coordinating ligands, provides such a hydrophobic environment [30], supporting a model in which ZQ_A_ occupies the active-site pocket rather than associating nonspecifically with the protein surface. This conclusion aligns with studies showing that 1:1 ZQ_A_–Zn species fluoresce at lower wavelength and increased intensity in liposomes compared to aqueous solution [10].

Consistent with conclusions from the fluorescence experiments, ICP-MS and HPLC/MS analyses of fractionated samples demonstrate the accumulation of a 1:1:1 ZQ_A_–Zn–NDM-1 complex following treatment with excess ZQ_A_ (Figure 4), while fluorescence lifetime measurements reveal simple decay dynamics indicative of a homogeneous local environment (Figure S8). In parallel, ^15^N–^1^H HSQC NMR titrations show pronounced chemical shift perturbations for residues near the active site only after the addition of sufficient ZQ_A_ to promote ternary complex formation. Importantly, these perturbations are localized, indicating that complex formation is accommodated through side-chain rearrangements that support the loss of one Zn^2+^ and a change in the coordination sphere of the other to accommodate the exogenous ZQ_A_ ligand. Modeling the EXAFS data supports such a mixed protein–probe coordination sphere around Zn^2+^.

The HADDOCK modeling of ZQ_A_^2−^ binding to mono-Zn NDM-1 provides a mechanistic framework that rationalizes the spectroscopic data. In the dominant binding pose, ZQ_A_ completes a near tetrahedral coordination sphere around the higher affinity Zn^2+^ while engaging in stabilizing π–π stacking interactions with active-site histidine residues. These interactions rigidify the ZQ_A_ scaffold and distort the *p*-tolyl group. The twisted position likely disrupts conjugation across ZQ_A_’s sulfonamide linkage, which would affect emission energy, while restriction of intramolecular motion is expected to suppress non-radiative decay pathways, thereby driving increased fluorescence intensity. Although these models are necessarily predictive, they are supported by independent experimental measurements and offer a coherent explanation for the fluorescence behavior of the ternary complex.

The binding of ZQ_A_ to form the ternary complex appears to be reversible and competitive as suggested by the enzyme inhibition experiments conducted with concentrations of ZQ_A_ and the nitrocefin substrate much higher than the enzyme. Prior work has demonstrated that mono-Zn NDM-1 retains β-lactamase activity although at a significantly slower rate than holo-NDM-1 [29]. Given these considerations, the measured IC_50_ value of 2.4 μM provides a reasonable approximation of the apparent binding affinity of ZQ_A_ to Zn–NDM-1 under these assay conditions. This estimate is biophysically plausible, accounting for loss of the weaker bound Zn^2+^ and coordination with the stronger bound Zn^2+^ [19,20], and in the range reported for ternary complexes with another Zn^2+^ fluorescent probe [31].

The work presented here demonstrates that the blue-shifted fluorescence emission of ZQ_A_ arises from the formation of a well-defined ZQ_A_–Zn–NDM-1 ternary complex, which positions the probe within a hydrophobic environment that affects both emission intensity and wavelength. The formation of this ternary complex is important to showcase the propensity of ZQ_A_ specifically, but small molecules more generally, to engage metalloproteins through specific, zinc-mediated interactions that are spectroscopically distinct from free metal–probe complexes. By delivering direct molecular and biophysical evidence to support prior models of ternary complex formation [12,15,32,33], this study underscores the importance of understanding the full scope and complexity of interactions accessible to chemical biology probes.

## 4. Materials and methods

### 4.1. General chemical and biochemical information

All reagents and solvents were purchased from commercial sources (Acros, Bio-Rad, Koptec, Millipore-Sigma, Oakwood Chemical, Santa Cruz Biotechnology, Zymo Research) and used without further purification. Aqueous solutions were prepared in ultrapure water from a Hydro PicoPure UV Plus water system.

### 4.2. Standard solution preparation

Buffers for protein purification were prepared by dissolving the appropriate amount of *N*-(2-hydroxyethyl)piperazine-*N*-2-ethanesulfonic acid (HEPES) sodium salt, sodium chloride, imidazole, and glycerol in ultrapure water. The solutions were cooled to 4 °C overnight, and the pH was adjusted using 12 M HCl or 50 wt% NaOH with a calibrated Oakton 550 pH probe. Tris-Gly-SDS 10× solution was purchased from Bio-Rad and diluted to 10% with ultrapure water for use in gel electrophoresis.

The following aqueous buffers were used during protein purification and other experiments. Wash Buffer—50 mM HEPES, 300 mM NaCl, 10 mM imidazole, 10 μM ZnSO_4_, 10% glycerol, 4 °C, pH 8.0; Elution Buffer—50 mM HEPES, 300 mM NaCl, 500 mM imidazole, 10 μM ZnSO_4_, 10% glycerol, 4 °C, pH 8.0; EK Cleavage Buffer—50 mM HEPES, 150 mM NaCl, 10% glycerol, 4 °C, pH 8.0; Storage Buffer—50 mM HEPES, 150 mM NaCl, 10% glycerol, 4 °C, pH 7.5.

Solutions of Zinquin free acid (ZQ_A_) were prepared by dilution from a 100 mM stock solution made by dissolving 5 mg of ZQ_A_ solid in DMSO.

### 4.3. Whole-plasmid sequencing

The strain *E. coli* BL21(DE3) containing the NDM-1_pET-30a(+) plasmid was grown overnight at 37 °C on LB agar plates containing 50 μg/mL kanamycin. A single colony was used to inoculate 5 mL of liquid LB broth with 50 μg/mL kanamycin and grown for 20 h at 37 °C and 200 rpm. Cells were harvested by centrifugation (4000 rcf, 5 min, RT) and resuspended in 0.5 mL of ultrapure water, and DNA was isolated using the Zymo Research Zyppy Plasmid Miniprep Kit. Purified DNA was sent to Plasmidsaurus for whole-plasmid Oxford Nanopore Technologies sequencing. An illustration of the open reading frame for the expressed NDM-1 fusion protein from NDM-1_pET-30a(+) is available in the Supporting Information (Figure S9). Plasmid sequencing results are available in the Supporting Data.

### 4.4. Expression, isolation, and purification of recombinant NDM-1

The strain *E. coli* BL21(DE3) containing a pET-30a(+) plasmid cloned with NDM-1, without its N-terminal signal sequence, was grown overnight at 37 °C on LB agar plates containing 50 μg/mL kanamycin. A single colony was used to inoculate 5 mL of liquid LB broth with 50 μg/mL kanamycin and incubated for 20 h at 37 °C and 200 rpm, for each 1.5 L growth. The 5 mL starter culture was decanted into 1.5 L of fresh liquid LB broth with 50 μg/mL kanamycin and grown to OD_600_ 0.5–0.6. Once at the appropriate OD_600_, 1.5 mL of 200 mM isopropyl β-D-1-thiogalactopyranoside (IPTG) and 1.5 mL of 500 mM ZnSO_4_ were added to induce expression of the NDM-1 fusion protein. Cells were incubated for 20 h at 18 °C and 200 rpm.

Cells were harvested by centrifugation (6000 rcf, 15 min, 4 °C) and pellets were resuspended in Wash Buffer (15 mL for every 1.5 L growth) containing Roche cOmplete Mini EDTA-free tablets (1 tablet/15 mL Wash Buffer) and lysozyme (1 mg/mL Wash Buffer). The resuspended cells were then incubated for 30 min at 4 °C on a conical vial rotator. Cells were lysed on ice using a Fisher Scientific FB120 sonicator at 30% amplitude cycles of 1 s “on” and 2 s “off” for a total “on” time of 6 min. Cell debris was separated by centrifugation (24,000 rcf, 25 min, 4 °C), and the clarified lysate was decanted into a fresh vial for FPLC purification.

The 6xHis_NDM-1 fusion proteins were isolated from the lysate by immobilized metal (nickel) affinity chromatography (IMAC) on an AKTA pure FPLC instrument at 4 °C using a Cube Biotech 5 mL INDIGO column. A step gradient of Elution Buffer in Wash Buffer and a flow rate of 1.0 mL/min were used. The gradient program included a loading sequence to load the entire volume of lysate, an initial hold at 0% Elution Buffer for 10 column volumes (CVs) to wash excess lysate, protein elution with a step increase to 50% Elution Buffer for 10 CVs, a step increase to 80% Elution Buffer for 10 CVs, a step increase to 100% Elution Buffer for 10 CVs, and re-equilibration back to 0% Elution Buffer for 10 CVs. The inline U9-L UV monitor was used to acquire a UV chromatogram at 280 nm. Samples containing the target protein as indicated by the UV chromatogram were combined, concentrated, and washed with EK Cleavage Buffer using a 20 mL 10K MWCO PES Pierce protein concentrator spin filter.

The 6xHis_NDM-1 fusion protein solution was diluted to 40 mL with EK Cleavage Buffer. The 6xHis tag was removed by incubating the fusion protein solution with 6xHis_Enterokinase (GenScript Z03004) at RT on a conical vial rotator. The reaction was monitored by denatured gel electrophoresis and HPLC/MS, using Bio-Gel P-6 size exclusion chromatography (SEC) spin columns to desalt and remove glycerol before injection.

The 6xHis peptides and 6xHis_Enterokinase were removed by batch-spin purification using five portions of 2 mL of 50% Cube Biotech INDIGO IMAC resin equilibrated with Wash Buffer. The protein reaction solution was decanted into a conical vial containing the equilibrated IMAC resin. The mixture was incubated for 1 h at 4 °C on a conical vial rotator. The resin was separated by centrifugation (500 rcf, 5 min, 4 °C). The supernatant was transferred to a conical vial containing another portion of fresh IMAC resin. The previous steps were repeated for all portions of the equilibrated IMAC resin. The protein solution was concentrated and washed with Storage Buffer using a 20 mL 10K MWCO PES Pierce protein concentrator spin filter. Protein solutions were quantified by Bradford assay, aliquoted, snap-frozen in liquid nitrogen, and stored at −80 °C. The typical yield of NDM-1 was 35 mg/L of LB induction media. Purified NDM-1, typically 5–15% Zn-loaded, was characterized by denatured gel electrophoresis, HPLC/MS, and SEC. Characterization results are available in the Supporting Information (Figures S10, S11, S13, S14 and S16).

To prepare Zn-loaded, holo-NDM-1, a solution of purified NDM-1 was mixed with 10 molar equivalents of ZnSO_4_ in Storage Buffer and then spun in a 10K MWCO Pierce protein concentrator spin filter at 14,000 rcf at 4 °C for 45 min. The retained sample was washed with Storage Buffer, and the spin-filter step was repeated twice more. Apo-NDM-1 was prepared in a similar manner but treated with 5 mM EDTA instead of ZnSO_4_. The protein concentration for apo- and holo-NDM-1 was quantified by Bradford assay and the Zn^2+^ concentration was quantified by ICPMS.

### 4.5. Expression, isolation, and purification of recombinant ^15^N-NDM-1

Previous protocols were referenced for expression, isolation, and purification [34–36]. Recipes for M9 salts and nutrients, ^15^N-ammonium chloride, and glucose are available in the Supporting Information.

The strain *E. coli* BL21(DE3) containing a pET-30a(+) plasmid cloned with NDM-1, without its N-terminal signal sequence, was grown overnight at 37 °C on LB agar plates containing 50 μg/mL kanamycin. A single colony was used to inoculate each of two 5 mL of liquid LB broth with 50 μg/mL kanamycin and grown for 20 h at 37 °C and 200 rpm. Each of the 5 mL starter cultures was decanted into a 1.5 L of fresh liquid LB broth with 50 μg/mL kanamycin and grown to OD_600_ 0.8–1.0. Once at the appropriate OD_600_, the flasks were removed from the incubator and set in a 4 °C cold room. Cells were harvested by centrifugation (6000 rcf, 15 min, 4 °C) and pellets were resuspended in 160 mL of M9 salts and nutrients. The cell suspension was poured into a flask with 500 mL of pre-warmed, 37 °C M9 salts and nutrients. The cells were incubated for 45 min at 37 °C and 200 rpm before 75 mL of 10 × ^15^N-ammonium chloride and 15 mL of 50× glucose were added, bringing the total volume to 750 mL (4×concentration of LB cell suspension). Immediately, 750 μL of 1 M IPTG and 750 μL of 500 mM ZnSO_4_ were added to induce expression of the ^15^N-NDM-1 fusion protein. Cells were incubated for 20 h at 18 °C and 200 rpm.

Protocols for cell lysis and purification of ^15^N-NDM-1 followed those in Section 4.4. The typical yield of ^15^N-NDM-1 was 40 mg/L of M9 induction media. Purified ^15^N-NDM-1 was characterized by denatured gel electrophoresis, HPLC/HRMS, and SEC. Characterization results are available in the Supporting Information (Figures S10, S12, S13, S15 and S17).

To form apo ^15^N-NDM-1, the protein solution was dialyzed at 4 °C against 4 × 500 mL of 100 mM Bis-Tris, 150 mM NaCl, 20 molar equivalents of EDTA, and pH 7; then 4 × 500 mL of 100 mM Bis-Tris, 150 mM NaCl, and pH 7. To form holo ^15^N-NDM-1, the protein solution was dialyzed at 4 °C against 4 × 500 mL of 100 mM Bis-Tris, 150 mM NaCl, 10 molar equivalents of ZnSO_4_, and pH 7; then 4 × 500 mL 100 mM Bis-Tris, 150 mM NaCl, and pH 7.

### 4.6. Bradford assay

The NDM-1 concentration was quantified by injecting 7.00 μL of NDM-1 solution into 293.0 μL of Pierce Bradford Plus Protein Assay Reagent. The solution was vortexed, and 200 μL was transferred to a single well in a 96-well plate. Absorbance at 600 nm was recorded on a PerkinElmer Victor^3^ V 1420 plate reader. A calibration curve of absorbance at 600 nm versus bovine serum albumin (BSA) concentration was prepared fresh to determine the concentration of the NDM-1 solution. The BSA standards of 1000, 750, 500, 250, 125, and 0 μg/mL in ultrapure water were prepared from ampules of 2 mg/mL of Pierce BSA standard and analyzed identically to the protein sample. The calibration curves had an *r*^2^ ≥ 0.95. The NDM-1 solutions were diluted as needed to be within the calibration curve range.

### 4.7. Denatured gel electrophoresis

The sample loading buffer was prepared with 9:1 4× Laemmli Sample Buffer (Bio-Rad):2-mercaptoethanol; 12.5 μL of sample loading buffer and 37.5 μL of the protein solution were mixed and incubated at 95 °C for 10 min. Bio-Rad Mini-PROTEAN TGX 4–20% precast gels were run in Tris-Gly-SDS buffer at 140 V loaded with 5–10 μL of protein sample solutions in each of the wells. A Bio-Rad Precision Plus Kaleidoscope ladder (5 μL) was run in adjacent wells as a protein-molecular-weight calibrant. Gels were stained with a Coomassie blue solution (recipe: 450:450:50 mL ultrapure water:methanol:acetic acid, 0.5 g Coomassie blue) for 30 min, destained (recipe: 450:450:50 mL ultrapure water:methanol:acetic acid), and imaged on an Invitrogen iBright 1500. Images of gels are available in the Supporting Information (Figures S10 and S13).

### 4.8. HPLC/MS characterization

The HPLC/MS analyses were acquired on an Agilent 1260/6460 instrument. Positive-ion mass spectra were acquired in full-scan mode over the range of 100–2500 m/z using the following source parameters: gas temperature 300 °C, gas flow 5 L/min, nebulizer pressure 45 psig, VCap 3500 V, and fragmentor voltage 135 V. The HPLC separations were achieved on a Phenomenex Luna C18(2) column (1 × 50 mm, 3 μm, 100 Å) using a linear gradient of mobile phase B in A, a flow rate of 0.5 mL/min, and a column temperature of 40 °C. Mobile phase A was prepared by combining 400 mL ultrapure water with 12 mL methanol and 1.2 mL formic acid. Mobile phase B was prepared by combining 400 mL acetonitrile with 12 mL ultrapure water and 1.2 mL formic acid. The gradient program included an initial hold at 0% solvent B for 2 min, linear increase to 60% solvent B from 2 to 9 min, linear increase to 100% B from 9 to 10 min, a hold at 100% B from 12 to 14.5 min, linear decrease to 0% B from 12 to 14.5 min, and final hold at 0% B from 14.5 to 15 min for a total run time of 15 min. In addition to MS detection, the DAD was used to acquire a UV chromatogram at 220 nm, 254 nm, and 280 nm. Samples were analyzed using a 2 μL injection volume. Data were exported from Agilent MassHunter and visualized in Microsoft Excel. The MS results are available in the Supporting Information (Figures S11, S12, S14 and S15).

The HPLC/HRMS analyses were acquired on an Agilent 1200/6224 instrument. The mass spectrometer was equipped with a Dual ESI source, and accurate mass data were obtained by internal calibration (reference ions 121.050873 and 922.009798 m/z) using a secondary nebulizer to continuously deliver the reference solution. Positive-ion mass spectral data were acquired in full-scan mode over the range of 100–3200 m/z using the following source parameters: gas temperature 325 °C, gas flow 11 L/min, nebulizer pressure 33 psig, VCap 3500 V, and fragmentor voltage 220 V. Mass spectral data were acquired in profile mode, and raw spectra were deconvoluted using Agilent BioConfirm. The HPLC separations were achieved on a Phenomenex Kinetex EVO C18 column (3 × 100 mm, 2.6) using a linear gradient of mobile phase B in A, a flow rate of 0.5 mL/min, and a column temperature of 40 °C. Mobile phases A and B were prepared as described above. The gradient program included an initial hold at 30% solvent B for 0.5 min, followed by a linear increase to 60% solvent B from 0.5 to 18 min, a hold at 100% solvent B from 18 to 19 min, and re-equilibration back to 30% B for a total run time of 25 min. In addition to MS detection, the DAD was used to acquire a UV chromatogram at 220 nm, 254 nm, and 280 nm. Samples were analyzed using a 1–5 μL injection volume. The HRMS results are available in the Supporting Information.

### 4.9. Size exclusion chromatography

The SEC analyses were acquired on an AKTA pure FPLC instrument at 4 °C. An inline U9-L UV monitor was used to acquire a UV chromatogram at 280 nm. Separations were achieved on a Cytiva Superdex 75 Increase 10/300 GL column by isocratic elution with SEC Run Buffer at a flow rate of 0.4 mL/min. Run Buffer for native NDM-1 consisted of 50 mM HEPES, 100 mM NaCl, 5% glycerol, 4 °C, and pH 7.5. Run Buffer for ^15^N-NDM-1 consisted of 100 mM Bis-Tris, 150 mM NaCl, 4 °C, and pH 7.0. Raw data files were exported from Cytiva UNICORN and visualized in Microsoft Excel. The SEC traces are available in the Supporting Information (Figures S16 and S17).

### 4.10. NDM-1 activity assay

A ZQ_A_ stock solution (100 mM in DMSO) was diluted to 4× the highest tested concentration with 50 mM HEPES (pH 7.5). A final volume of 200 μL was used for all wells in a 96-well microplate, where 100 μL 2× serial microdilutions were performed across the long axis of the plate. Holo-NDM-1 was diluted to 10 nM in 20 mL of 50 mM HEPES (pH 7.5) and 100 μL was added to the appropriate wells in the prepared 96-well microplate (5 nM NDM-1). The 96-well plate was then incubated at RT for 25 min. Each microplate had an entire row of no treatment control and a positive control 2× serial microdilution of pyrithione (20 mM in DMSO). A nitrocefin stock solution (5 mg/mL in DMSO) was diluted to 400 μM with 50 mM HEPES (pH 7.5) to prepare 1.5 mL. Rapidly, 10 μL portions of the 400 μM nitrocefin solution were injected into each well. Using a PerkinElmer Victor^3^ V 1420 plate reader, the change in nitrocefin absorbance at 490 nm was recorded at 5 min intervals for 30 min to determine the NDM-1 reaction rate. The relative reaction rate was normalized to the inhibitor-lacking control for the given experiment. For nonlinear regression, a Levenberg–Marquardt estimation method was used with a sum-of-squares convergence of 1 × 10^−8^ and parameter convergence of 1 × 10^−8^.

### 4.11. Fluorescence spectroscopy

Fluorescence emission spectra for ZQ_A_-containing samples (400–600 nm, *λ*_ex_ = 370 nm) were recorded in quartz cuvettes on an Edinburgh Instruments (Livingston, UK) FS5 Spectrofluorometer, with parameters set at 1 nm slit widths, a 0.1 s dwell time, and three averaged scans. For titration experiments, aliquots of ZQ_A_ were added to solutions of apo-NDM-1, holo-NDM-1, and ZnSO_4_ (10 μM each) in Storage Buffer (diluted to 30 mM HEPES).

### 4.12. Fractionation sample preparation

The fractionation sample preparation workflow is shown schematically in Figure S1. Samples were prepared by adding increasing equivalents of ZQ_A_ to 20 μM holo-NDM-1 in Storage Buffer (diluted to 37.5 mM, pH 7.5). The samples were incubated by shaking at 200 rpm at RT for 30 min and then spun in 10K MWCO Pierce PES protein concentrator spin filters at 14,000 rcf (4 °C, 15 min). The compounds may be able to form nonspecific, hydrophobic interactions within the PES membrane, a limitation of PES known within pharmaceutical sciences [37]. This adsorption is suppressed by the addition of 150 mM NaCl, which is present in the Storage Buffer [37]. The retained sample was washed with 10× volumes of Storage Buffer, and the spin-filter concentration was repeated for a total of three times. The eluted flow-through from each spin cycle was kept for additional analysis. The resulting retained samples were extracted with a pipette and diluted to a total volume of 200 μL with Storage Buffer. Samples were prepared in quadruplicate. Control samples with varying molar equivalents of EDTA with holo-NDM-1 were prepared in the same manner. Each retained and eluted sample was analyzed for protein, ZQ_A_, and Zn^2+^ concentrations as well as fluorescence emission and UV–vis absorbance. The protein concentration was measured by Bradford assay. The Zn^2+^ and ZQ_A_ contents were quantified by ICP-MS and HPLC/MS, respectively. The UV–vis spectra were scanned from 200 to 600 nm on a Varian Cary 50 UV–vis Spectrophotometer after taking a baseline and zero scan. A control titration of ZnSO_4_ into a solution of 20 μM ZQ_A_ in Storage Buffer was used to compare with the fractions.

### 4.13. Quantification of Zn^2+^ by ICP-MS

The ICP-MS analyses were acquired on an Agilent 7900 ICP-MS in He mode. Both the retained and eluted samples were diluted into a solution of 2% (v/v) trace metal grade nitric acid with 0.5% (v/v) trace metal grade hydrochloric acid spiked with 20 ppb ^72^Ge, ^89^Y, and ^115^In as internal standards. Elements were measured under He atmosphere (collision cell) to reduce polyatomic interferences with a He gas flow rate of 4.3 mL/min. Data were quantified using weighed, serial dilutions of multi-element standards (CLMS-2AN [SPEX CertiPrep, CL51-042CRYA]). A premixed drinking water standard (CRM-TMDW-A from High Purity Standards) was used as an initial calibration verification. Internal standards ^72^Ge, ^89^Y, and ^115^In were kept within±10% of the expected value. Each sample was analyzed seven times on the ICP-MS with four biological replicates.

### 4.14. Quantification of ZQ_A_ by HPLC/MS

The ZQ_A_ concentrations in the retained fractionation samples were quantified by HPLC/MS using an Agilent 1260/6460 instrument. The HPLC mobile phase preparation and gradient program were identical to those described in Section 4.8. For the first 2 min, flow from the HPLC was routed to the waste to avoid the buildup of buffer components in the MS. A complete sequence with standard curve development, injection washes, quality-control standards, and samples was performed. A standard curve of the integrated extracted ion chromatogram versus ZQ_A_ concentration was developed from 2 μL injections of 0.125–10 μM ZQ_A_ in 10% Storage Buffer (Figure S5). Separate 4 μM quality-control samples were quantified every sixth sample injection, and flanked by 10 μL ultrapure water injections, to verify instrument performance across the sample sequence. Quality-control samples were within 7% error of the standard curve. Retained and eluted fractionation samples were diluted 10× with ultrapure water and injected (2 μL) into the HPLC/MS to quantify ZQ_A_ concentration. Raw data were exported from Agilent MassHunter and processed in Microsoft Excel. The chromatogram for ZQ_A_ standards, ZQ_A_ standard curve, and the chromatogram for quality-control samples are available in the Supporting Information (Figures S4–S6).

### 4.15. Analysis of Zn^2+^ -binding ligands by EXAFS

Samples containing NDM-1, Zn^2+^, and ZQ_A_ were prepared by incubating 5 molar equivalents of ZQ_A_ to 0.7 mM holo-NDM-1 in Storage Buffer (37.5 mM HEPES) at pH 7.4 for 30 min before being washed in a Pierce concentrator as described in Section 4.13; both the retained and eluted samples were analyzed. Samples of mono-Zn NDM-1 were prepared by mixing 0.7 mM apo-NDM-1 with 1 molar equivalent of ZnSO_4_. A sample of 1 mM [Zn(ZQ_A_)_2_]^2−^ was prepared separately. Samples were injected with 20% ethylene glycol into a plastic cuvette with a tape window and frozen in liquid nitrogen.

The Zn K-edge (9.66 KeV) EXAFS and X-ray absorption near edge spectra were collected at the Stanford Synchrotron Radiation Lightsource on beamline 7–3 with an Si 220 monochromator, in fluorescence mode using a high-count rate Canberra 30-element Ge array detector. A Cu filter and a Soller slit were placed in line with the detector to attenuate the elastic scatter peak. For energy calibration, a Zn^2+^ foil was placed between the second and third ionization chambers. Six scans of a buffer blank were averaged and subtracted from the raw data to produce a flat pre-edge and remove any residual Cu fluorescence. Data reduction and background subtraction were carried out using the EXAFSPAK suite [38], and the data were inspected for dropouts and glitches before averaging. The EXCURVE program was used for spectral simulations.

### 4.16. ^15^N–^1^H HSQC NMR titrations

The apo or holo ^15^N-NDM-1 solutions (100 mM Bis-Tris, 150 mM NaCl, 4 °C, pH 7.0) were concentrated to 200 μM or 1000 μM using 10K MWCO PES Pierce protein concentrator spin filters. The concentrated protein solution (500 μL) and D_2_O (50 μL) were added to an NMR tube and mixed by centrifugation and a 9″ NMR pipette. The ZQ_A_ was dissolved in DMSO-d_6_ at a concentration of 100 mM. This solution was spiked with a molar equivalent of NaOH and diluted with 100 mM Bis-Tris, 150 mM NaCl, 4 °C, and pH 7.0 to 50× molar equivalents of ^15^N-NDM-1. The ZQ_A_ additions were made in 10 μL portions per equivalent, mixing by centrifugation and a 9″ NMR pipette. Similarly, 50× molar equivalents of ZnSO_4_ dissolved in H_2_O was used to form mono-Zn, and holo ^15^N-NDM-1 during a titration. The ZQ_A_ titration of mono-Zn ^15^N-NDM-1 was performed by first adding and mixing 10 μL of 50× molar equivalents of ZnSO_4_ to apo ^15^N-NDM-1.

The 2D ^15^N–^1^H HSQC spectra were recorded at 298.1 K on a Bruker Avance III 600 MHz NMR spectrometer equipped with a ^1^H/^13^C/^15^N cryogenic probe. Mild precipitation of the protein was observed during the experiments, and significant precipitation was observed with 8 equiv of ZQ_A_. For this reason, data were not analyzed for samples with more than 4 equiv of ZQ_A_ added. For the 200 μM samples, HSQC experiments were recorded with spectral widths of 9.6 kHz for ^1^H and 2.4 kHz for ^15^N with 20 scans per increment and 128 data points in the indirect direction. For the 1000 μM sample, HSQC experiments were recorded with spectral widths of 9.6 kHz for ^1^H and 3.6 kHz for ^15^N with 8 scans per increment and 256 data points in the indirect direction. Additional spectra of the titrations are available in the Supporting Information (Figures S18–S20).

The change in chemical shift, Δδ, was calculated using the following equation, where $\Delta \delta_{\text{H}}$ and $\Delta \delta_{\text{N}}$ are changes in the chemical shift in the ^1^H and ^15^N dimensions, respectively:

$$\Delta \delta ={\left[{\left(\Delta \delta_{\text{H}}\right)}^{2}+{\left(\Delta \delta_{\text{N}}/5\right)}^{2}\right]}^{1/2}$$

Bruker NMR data, with peak annotations for holo ^15^N-NDM-1, are available in the Supporting Data.

### 4.17. Molecular dynamics simulations

The MD simulations were performed to provide a better representation of the NDM-1 and ZQ_A_ conformational flexibility and to more accurately characterize the solution structure of both prior to docking. The NDM-1 crystal structure PDB 5ZGR was used as the starting protein conformation [39], while ZQ_A_ (both 1^−^ and 2^−^ charge states) topology and parameter files were created through AMBER’s ANTECHAMBER and ACPYPE programs [40–42]. The MD simulations were performed with the GROMACS 2020.5 software package, which utilized 6+ CPU cores and one Nvidia Tesla K80 GPU [43]. For both ZQ_A_ molecules, initial MD simulations were performed in vacuo for 100 ns using the automatically generated ACPYPE scripts. The last structure from the in vacuo MD simulation was then utilized as the input structure for in-solution MD simulations.

In-solution, MD simulations were performed with the AMBER03 force field and the flexible simple point-charge water model. The initial structures were immersed in a periodic water box with a dodecahedral shape that extended 1 nm beyond the protein in all dimensions and were neutralized with counterions. Energy minimization was performed using the steepest descent algorithm, with a final maximum force below 100 kJ/mol/min (0.01 nm step size, cutoff of 1.2 nm for neighbor list, Coulomb interactions, and van der Waals interactions). After energy minimization, the system was equilibrated at 300 K and normal pressure for 1 ns. All bonds were constrained with the LINCS algorithm (cutoff of 1.2 nm for neighbor list, Coulomb interactions, and van der Waals interactions). After temperature stabilization, pressure stabilization was obtained by utilizing the v-rescale thermostat to hold the temperature at 300 K, and the Berendsen barostat was used to bring the system to 10^5^ Pa pressure. Production MD calculations (500 ns for NDM-1 and 100 ns for ZQ_A_ molecules) were performed under the same conditions, except that the position restraints were removed, and the simulation was run for 100 or 500 ns (cutoff of 1.1, 0.9, and 0.9 nm for neighbor list, Coulomb interactions, and van der Waals interactions, respectively). The root mean square deviation (RMSD of Cα for NDM-1 and all atoms for ZQ_A_ molecules) and radius of gyration (R_g_ of Cα for NDM-1 and all atoms for ZQ_A_ molecules) all confirmed stability and accuracy of the MD simulations by stabilizing after a 100 ns equilibration period (for NDM-1 and 10 ns for ZQ_A_ molecules), allowing for the analysis of the last 400 ns of the simulation for NDM-1 and 90 ns for ZQ_A_ molecules (Figure S20). GROMACS built-in and in-house scripts were used to analyze the MD simulation results [43]. All images were produced using Schrodinger’s PyMOL.

### 4.18. *Molecular docking of ZQ*_A_ *to NDM-1*

Default HADDOCK parameters were utilized locally throughout the docking procedure with the following exceptions: noecv = False, rotate180_0 = False, crossdock = True, and calcdesolv = True [44,45]. The ZQ_A_ molecules were targeted at NDM-1. Specifically, these residues were targeted: Leu 65, Met 67, Asp 124, Glu 152, and Gly 222 defined as active residues (directly involved in the interaction); Pro 68, Gly 69, Phe 70, Gly 71, Trp 93, Gln 123, Gly 127, Gly 153, Val 155, Ser 191, Gly 219, and Asp 223 defined as passive residues (indirectly involved in the interaction). For both charge states of ZQ_A_, all parts of the molecules were defined as active. Additionally, for the ZQ_A_ dianion docking to NDM-1, the quinoline and sulfonamide nitrogens were targeted directly at the Zn^2+^ ion with an initial distance from Zn^2+^ set to 2.0 Å and the ability to explore up to 4.0 Å but no closer than 2.0 Å. Ensembles created from the MD simulations above (11 structures for each) were used as starting structures for the docking procedure. The docking procedure consisted of the creation of 1000 complex structures for the first iteration (rigid docking), and 200 complex structures were generated for each subsequent iteration (semi-flexible docking and water refinement). A “fraction of common contact” clustering analysis was performed by defining the highest percentage of common contacts that contain at least 80% of the docked complexes. The structures in the clusters were scored locally using FireDock [46,47] and plotted in a boxplot to determine statistical outliers and median score through R-Studio. The structures that represented the median score for the highest populated cluster were then visualized with Schrodinger’s PyMOL to ensure the integrity of the complex formed.

## Supplementary Material

Supporting Information

Supplementary Data

Supporting information for this article is available on the journal’s website under https://doi.org/10.5802/crchim.482 or from the author. Fractionation scheme, additional fluorescence spectra, quantitative HPLC/MS, XAS, fluorescence decay with radiative rate, recipes for M9 salts and nutrients and M9 media for ^15^N labeling, characterization of NDM-1, additional ^15^N–^1^H HSQC NMR spectra, and MD simulation convergence.

## Figures and Tables

**Figure 1.**
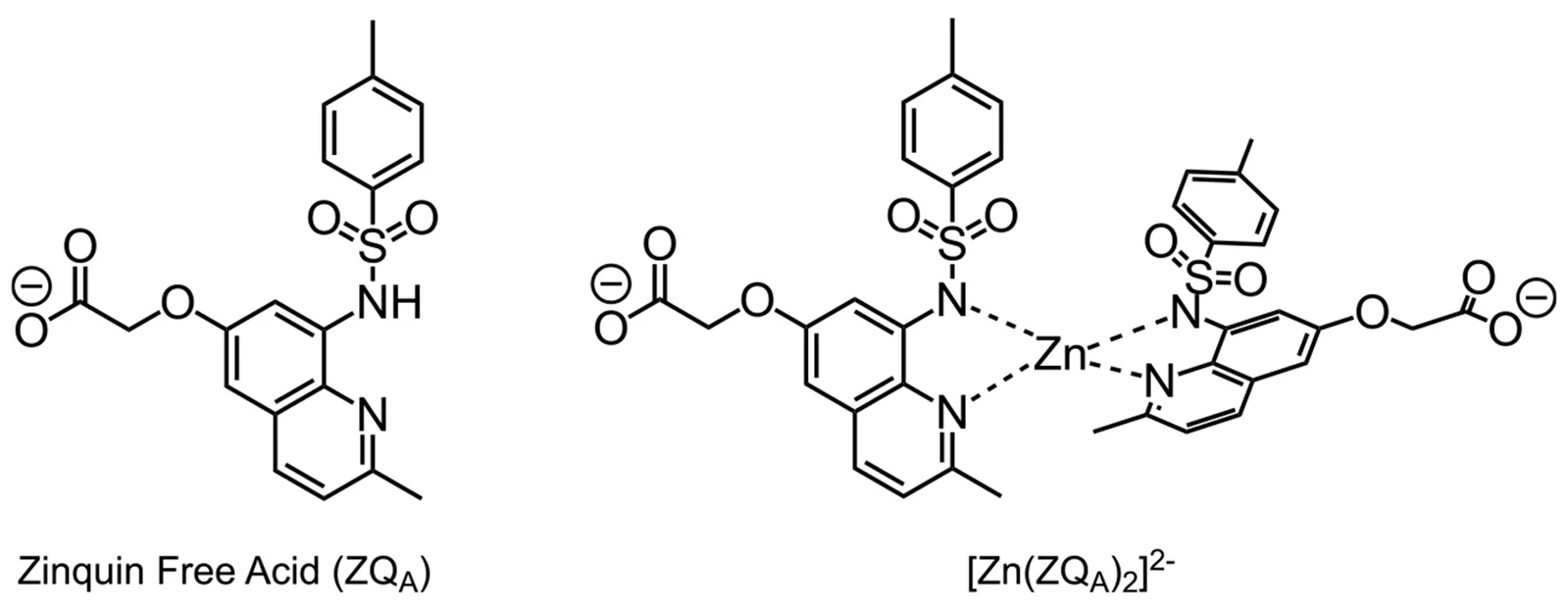
Chemical structures of ZQ_A_ and its [Zn(ZQ_A_)_2_]^2−^ complex.

**Figure 2.**
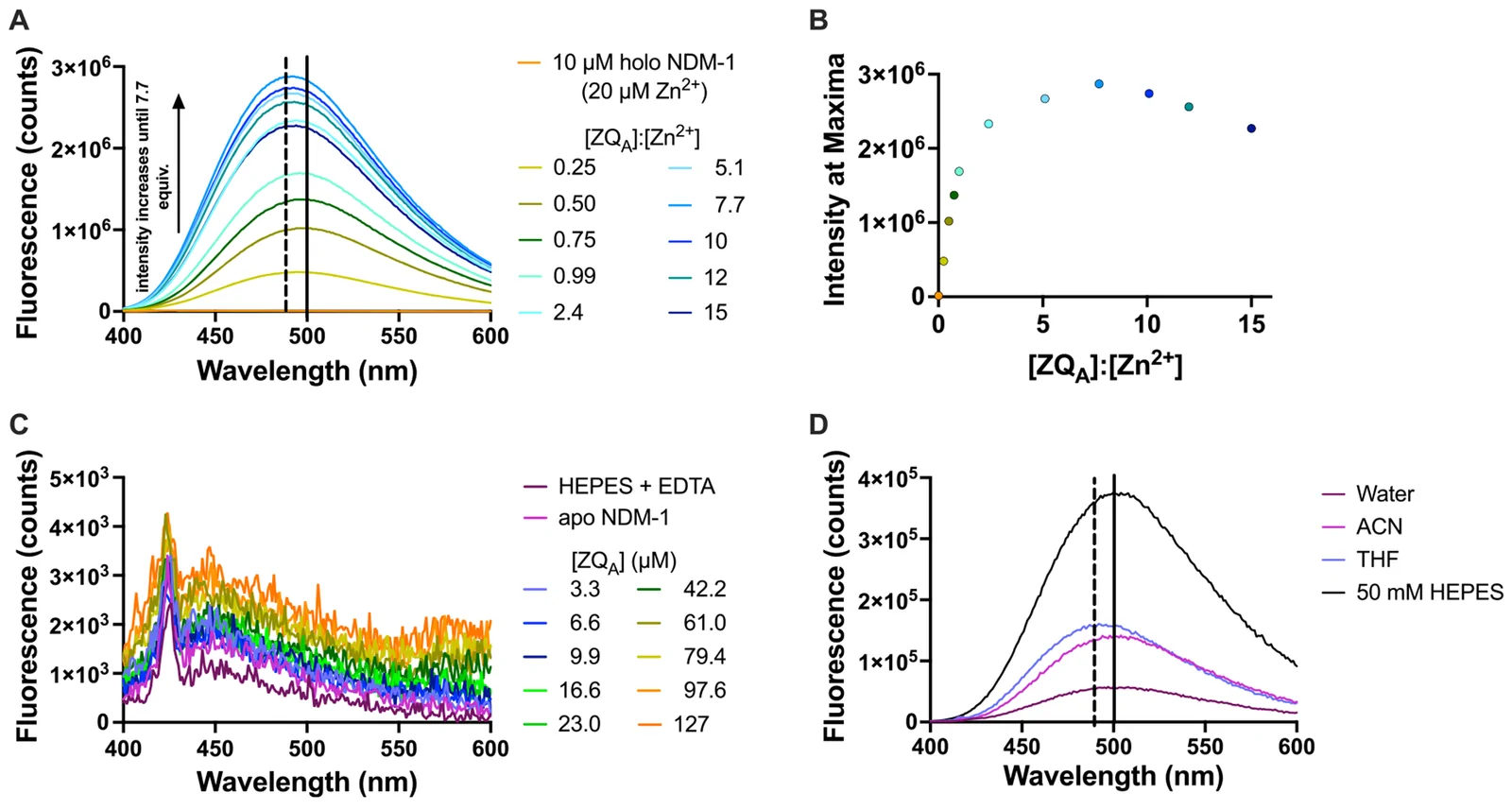
Blue shift in fluorescence does not occur in the absence of NDM-1. (A,B) Titration of ZQ_A_ into 10 μM holo-NDM-1 in protein Storage Buffer (37.5 mM HEPES, 150 mM NaCl, 10% glycerol, pH 7.5) reveals a blue shift and intensity increase for λ_max_ as a function of added ZQ_A_. (C) No fluorescence signal above noise appears for ZQ_A_ titrated into solutions of apo-NDM-1 with 2 mM EDTA to chelate trace Zn^2+^. (D) Fluorescence spectra of 10 μM [Zn(ZQ_A_)_2_]^2−^ in different solvent systems. Vertical black lines at 500 nm and dashed black lines at 488 nm distinguish fluorescence shifts in (A) and (D). Spectra are representative; additional trials available in Supporting Information Figure S2.

**Figure 3.**
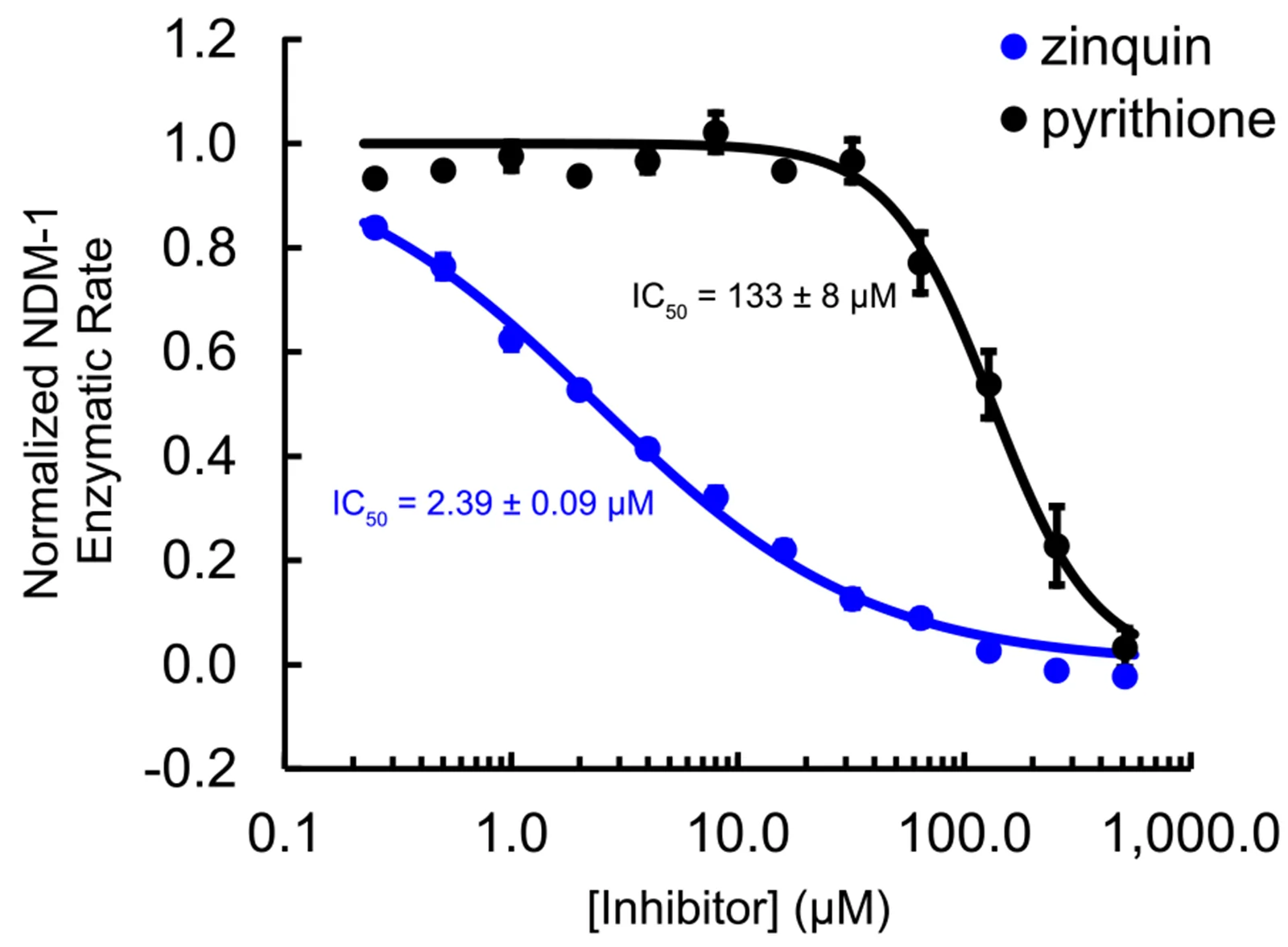
Enzymatic activity of NDM-1 is inhibited by ZQ_A_. Dose–response curves of NDM-1 activity for ZQ_A_ (blue) and pyrithione (black). Points and error bars are mean ± SEM from three experimental replicates of five technical replicates (*N* = 15). Fitted lines are logistic functions determined from nonlinear regression.

**Figure 4.**
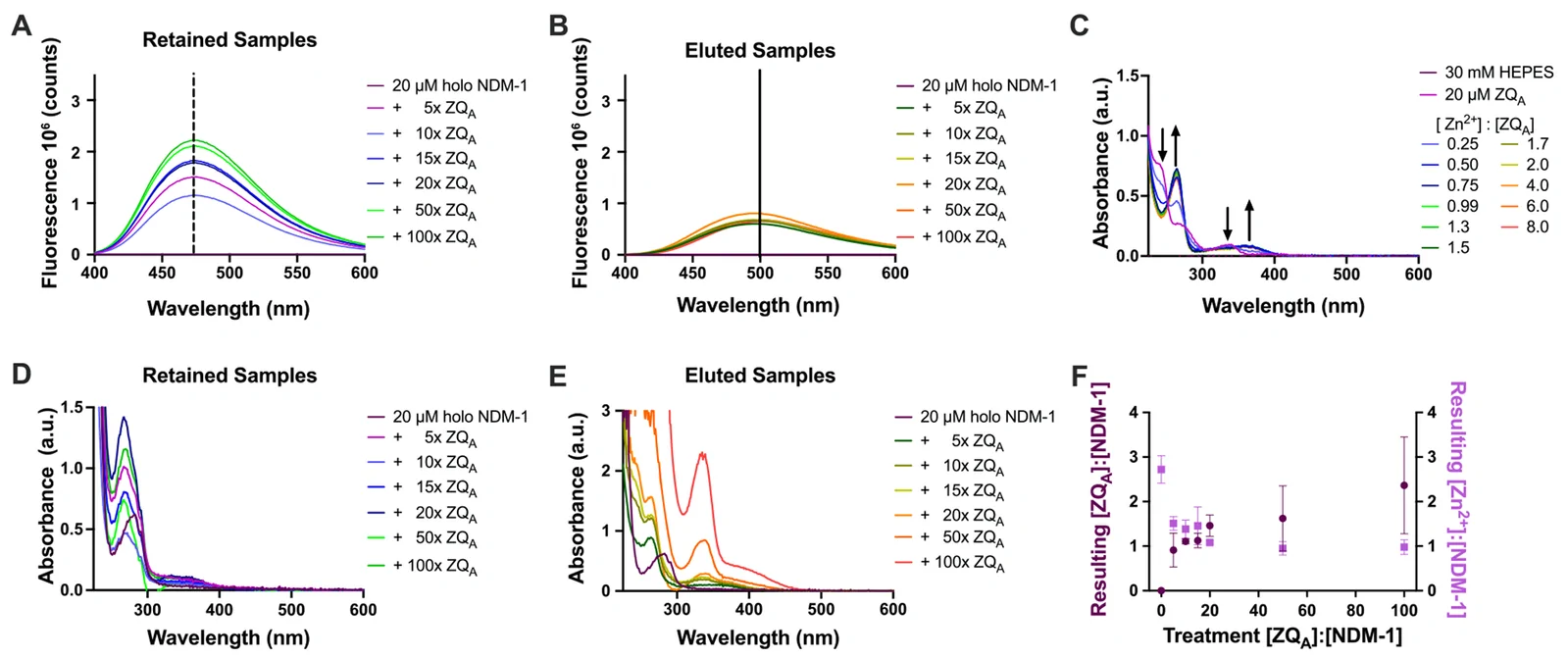
Fractionation of samples treated with different ratios of ZQ_A_ supports a 1:1:1 ternary complex formation. Fluorescence spectra of (A) retained and (B) eluted samples show λ_max_ at 473 nm and 500 nm, respectively. (C) UV–vis of ZQ_A_ upon titration with ZnSO_4_ in Storage Buffer (pH 7.5) shows that peaks for free ZQ_A_ at 240, 273, and 336 nm decrease as peaks at 264 and 360 nm increase upon Zn^2+^ complexation. UV–vis of the (D) retained and (E) eluted samples. (F) Quantitative determination of [ZQ_A_] (by HPLC/MS, left axis, dark purple circles) and [Zn^2+^] (by ICP-MS, right axis, light purple squares) relative to [NDM-1] (by Bradford assay) for the retained fractionation samples. A ternary species containing ZQ_A_, Zn^2+^, and NDM-1 in a 1:1:1 ratio is evident for samples treated with 5–20 molar equivalents of ZQ_A_. Spectra in (A,B,D,E) are representative of three replicates with additional trials shown in Supporting Information Figure S3; data points in (F) represent the average of four replicates with ± SEM.

**Figure 5.**
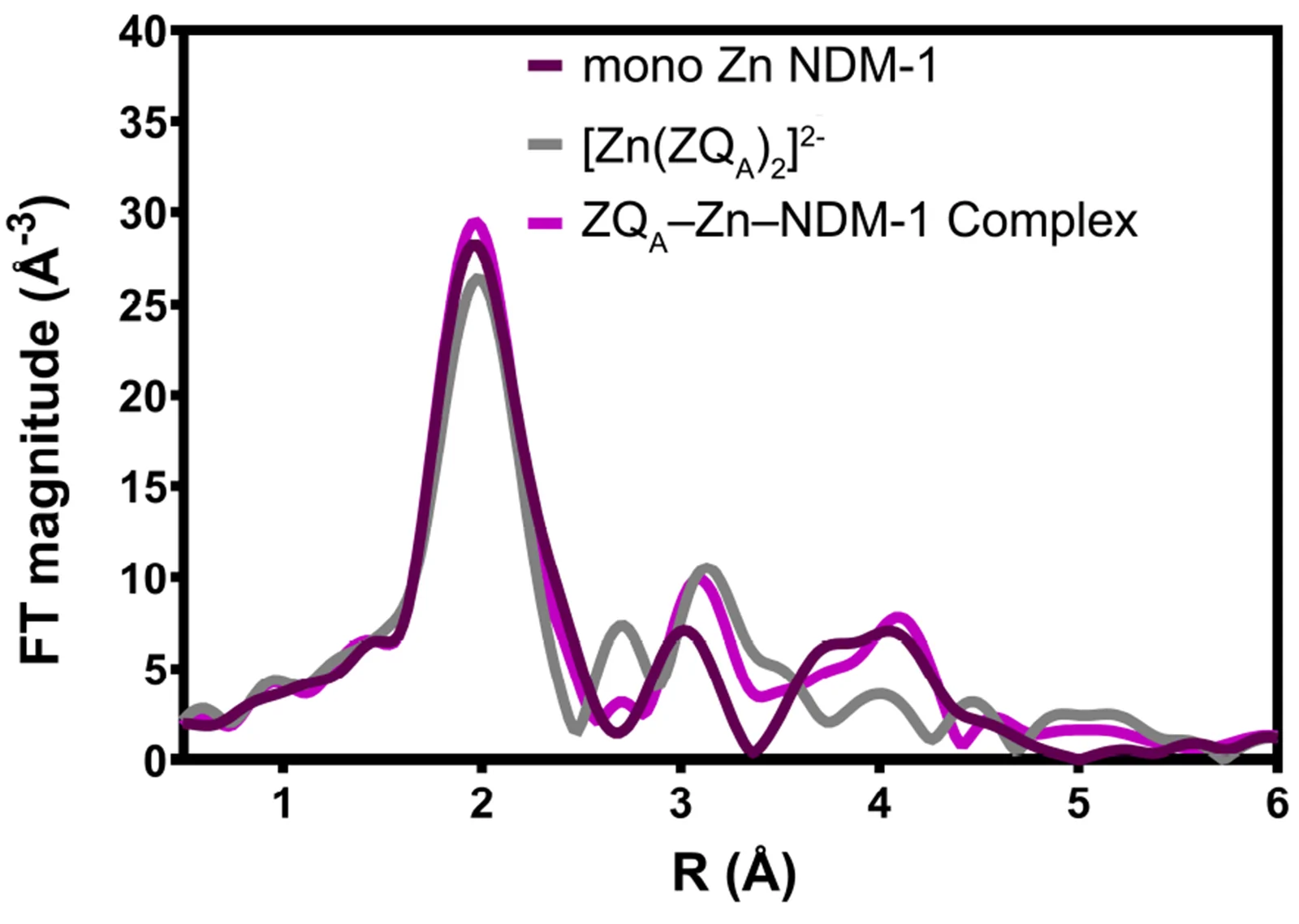
Overlay of Fourier-transformed Zn^2+^ K-edge EXAFS spectra for mono-Zn NDM-1 (dark purple), [Zn(ZQ_A_)]^2−^ (gray), and spin-filtered holo-NDM-1 following treatment with 5 equivalents of ZQ_A_ (light purple). The shared spectral features are consistent with mixed protein–ligand Zn^2+^ coordination.

**Figure 6.**
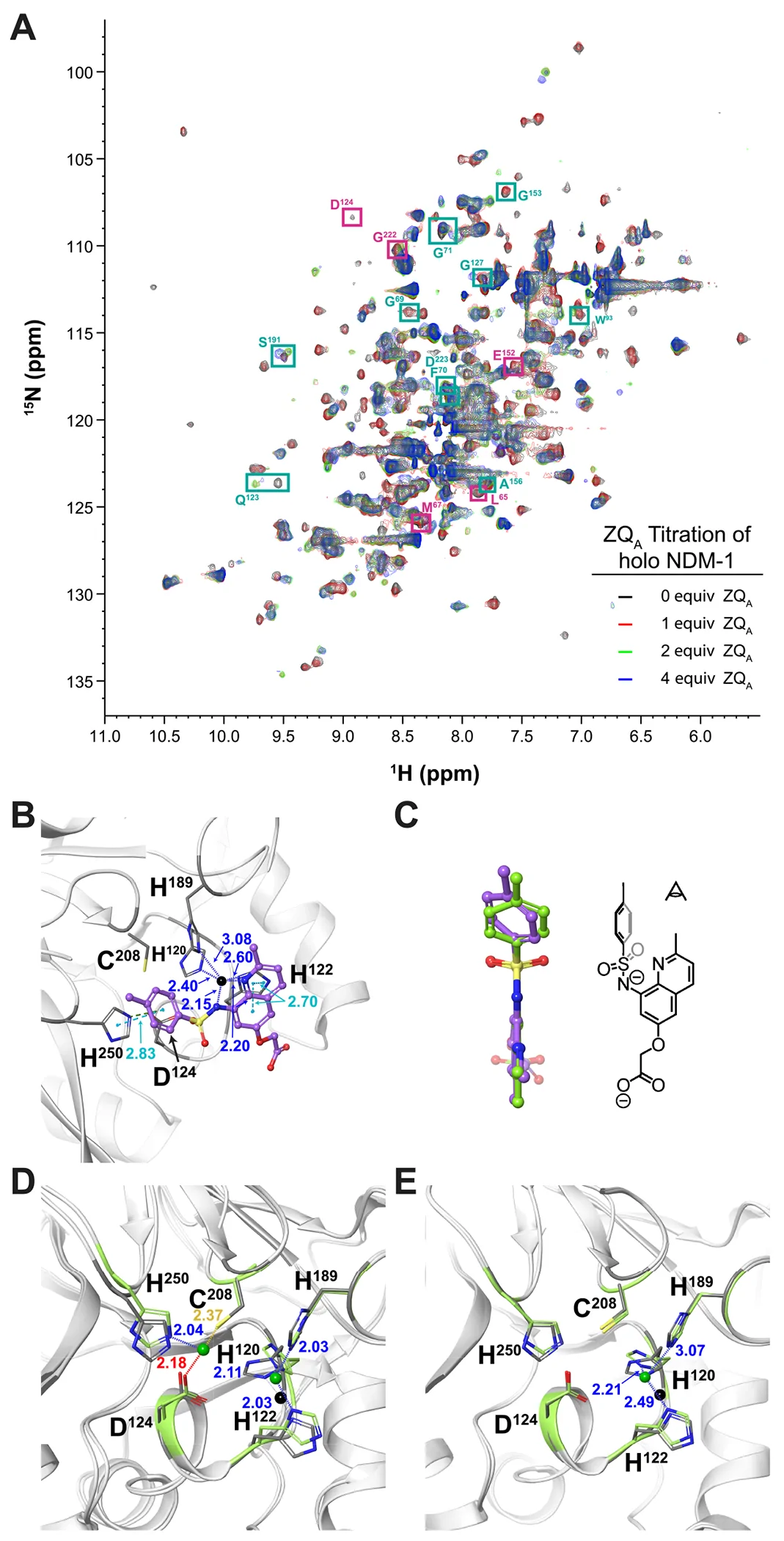
^15^N–^1^H HSQC NMR confirms ZQ_A_ forms a ternary complex with NDM-1. (A) Cross-peaks acquired by ^15^N–^1^H HSQC NMR of holo ^15^N-NDM-1 titrated with ZQ_A_. Addition of 1 equiv of ZQ_A_ results in minimal chemical shift perturbation, consistent with the initial portion of ZQ_A_ stripping Zn^2+^ from NDM-1 to form mono-Zn NDM-1. Once 2 equiv of ZQ_A_ has been added, strong chemical shift perturbations of residues around the active site indicate a molecular interaction with ZQ_A_. Disappearing cross-peaks labeled in magenta and cyan are directly and indirectly interacting residues, respectively. (B) HADDOCK model of ZQ_A_^2−^ bound to mono-Zn NDM-1. Quinoline and sulfonamide nitrogens of ZQ_A_^2−^ chelate to zinc to form a ternary complex. Cyan dashed line represents hydrophobic π–π stacking interactions of ZQ_A_ with H^250^ and H^122^. Distances between atoms in Å. Protein backbone shown with gray ribbon, white = hydrogen, gray = carbons of NDM-1, purple = carbons of ZQ_A_^2−^, blue = nitrogen, red = oxygen, yellow = sulfur, and black = zinc. (C) Alignment of ZQ_A_^2−^ from ternary complex (purple) vs geometry-optimized ZQ_A_^2−^ alone in water (green) to highlight distortion of the *p*-tolyl moiety in the ternary complex. Viewing angle of 3D overlay shown with skeletal structure. Alignment of NDM-1 active site from ZQ_A_ ternary complex with (D) holo-NDM-1 (PDB: 3Q6X) and (E) mono-Zn NDM-1 (PDB: 3SFP). For previously reported NDM-1 structures in (D) and (E), light green = carbon and green = zinc; distances between atoms in Å are shown.

## Data Availability

Plasmid sequencing results, Bruker NMR data, peak annotations for holo ^15^N-NDM-1, and mono-Zn NDM-1:ZQ_A_ HADDOCK structure are available for download, free of charge, from the Duke University Research Data Repository at https://doi.org/10.7924/r4xg9wb2w.

## Abbreviations

apo-NDM-1: NDM-1 with 0 equiv of Zn^2+^
BSA: Bovine serum albumin
EDTA: Ethylenediaminetetraacetic acid
EXAFS: Extended X-ray absorption fine structure
HADDOCK: High ambiguity driven protein–protein DOCKing
HEPES: 4-(2-hydroxyethyl)piperazine-1-ethanesulfonic acid
holo-NDM-1: NDM-1 with 2 equiv of Zn^2+^ (Zn_2_NDM-1)
HPLC/MS: High-performance liquid chromatography/mass spectrometry
HSQC: Heteronuclear single quantum coherence
IC_50_: Concentration that inhibits protein to 50% of basal activity
ICP-MS: Inductively coupled plasma mass spectrometry
*λ*_max_: Maximum fluorescence emission
MD: Molecular dynamics
mono-Zn: NDM-1 with 1 equiv of
NDM-1: Zn^2+^(Zn_1_NDM-1)
NMR: Nuclear magnetic resonance
PAR: 4-(2-pyridylazo)resorcinol
SEM: Standard error of the mean
THF: Tetrahydrofuran
TSQ: 6-methoxy-8-*p*-toluene sulfonamide quinolone
UV–vis: Ultraviolet–visible spectroscopy
XAS: X-ray absorption spectroscopy
ZQ: Zinquin
ZQ_A_: Zinquin free acid; 2-((2-methyl-8-((4-methylphenyl)sulfonamido) quinolin-6-yl)oxy)acetic acid
ZQ_A_^2−^: Zinquin dianion; (6-(carboxylatomethoxy)-2-methylquinolin-8-yl)(tosyl)amide
ZQ_EE_: Zinquin ethyl ester; ethyl 2-((2-methyl-8-((4 methylphenyl)sulfonamido) quinolin-6-yl)oxy)acetate
