# Supplementary material for: Mechanistic insights into protein-mediated Zinquin fluorescence
Source: C R Chim. Author manuscript; Available in PMC 2026 Nov 16. (PMC13771970; doi:10.5802/crchim.482)
Supplement: Supplementary file 1 [file NIHMS2221522-supplement-Supporting_Information.pdf]

Supporting Information

Mechanistic Insights into Protein-Mediated Zinquin Fluorescence

Natalie B. Schulte*[,1], Addison M. Duda*[,1], Sara M. Tuck[1], Kelly N. Chacón[2], Benjamin G. Bobay[1], Michael J. Therien[1], Andrew M. Dale[1], and Katherine J. Franz**[,1]

[1] *Department of Chemistry, Duke University, 124 Science Drive, Durham, NC 27710, USA*
[2] *Department of Chemistry, Reed College, Portland, OR 97202, USA*

*Authors contributed equally to this work
**Corresponding author e-mail: katherine.franz@duke.edu

Table of Contents

Page

Fractionation Scheme ____ 2

Additional Fluorescence Spectra ____ 3

Quantitative HPLC/MS ____ 5

XAS Fitting ____ 7

Fluorescence Decay with Radiative Rate ____ 9

Recipes for M9 Salts and Nutrients and M9 Media ____ 10

Characterization of Recombinant NDM-1 ____ 11

^{15}N-^{1}H HSQC NMR Titrations ____ 16

MD Simulations ____ 19

Fractionation Scheme

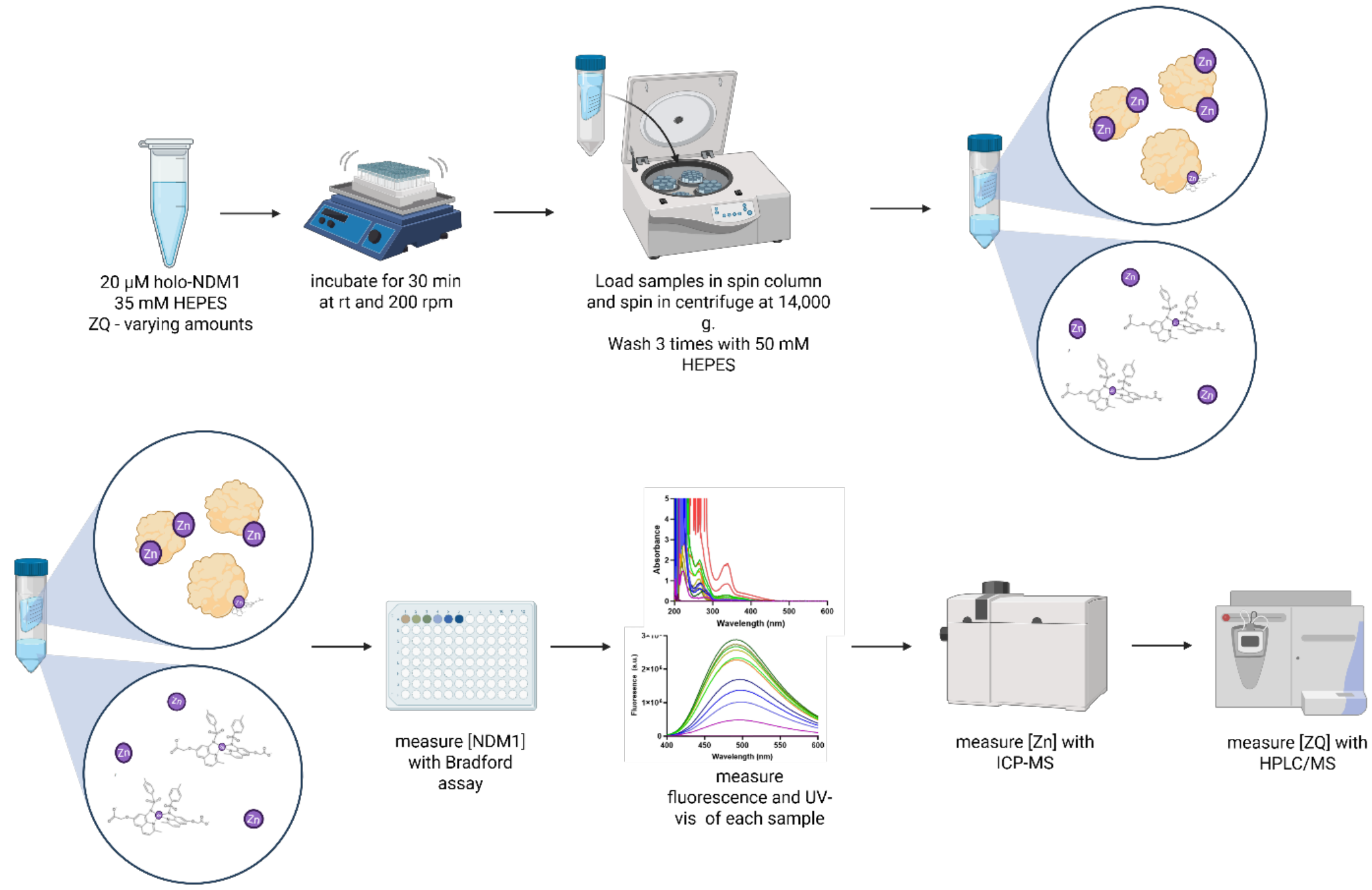

Figure S1. Workflow for fractionation sample prep.

Additional Fluorescence Spectra

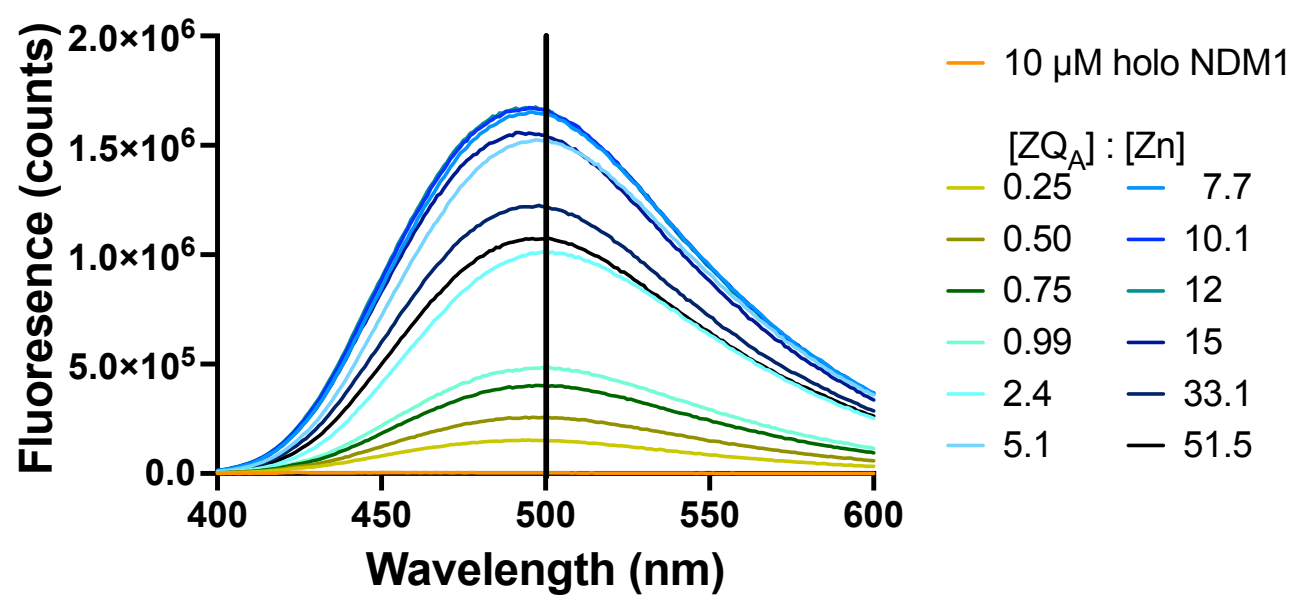

Figure S2. Additional fluorescence spectra for ZQ_A titration in holo NDM-1. ZQ_A was titrated into a solution of 10 μM holo NDM-1 (NDM-1 with 20 μM Zn bound) in Storage Buffer (37.5 mM HEPES). Blue shift from expected λ_{max} of 500 nm to a λ_{max} of 488 nm was observed after 1 ZQ_A:1 Zn was reached.

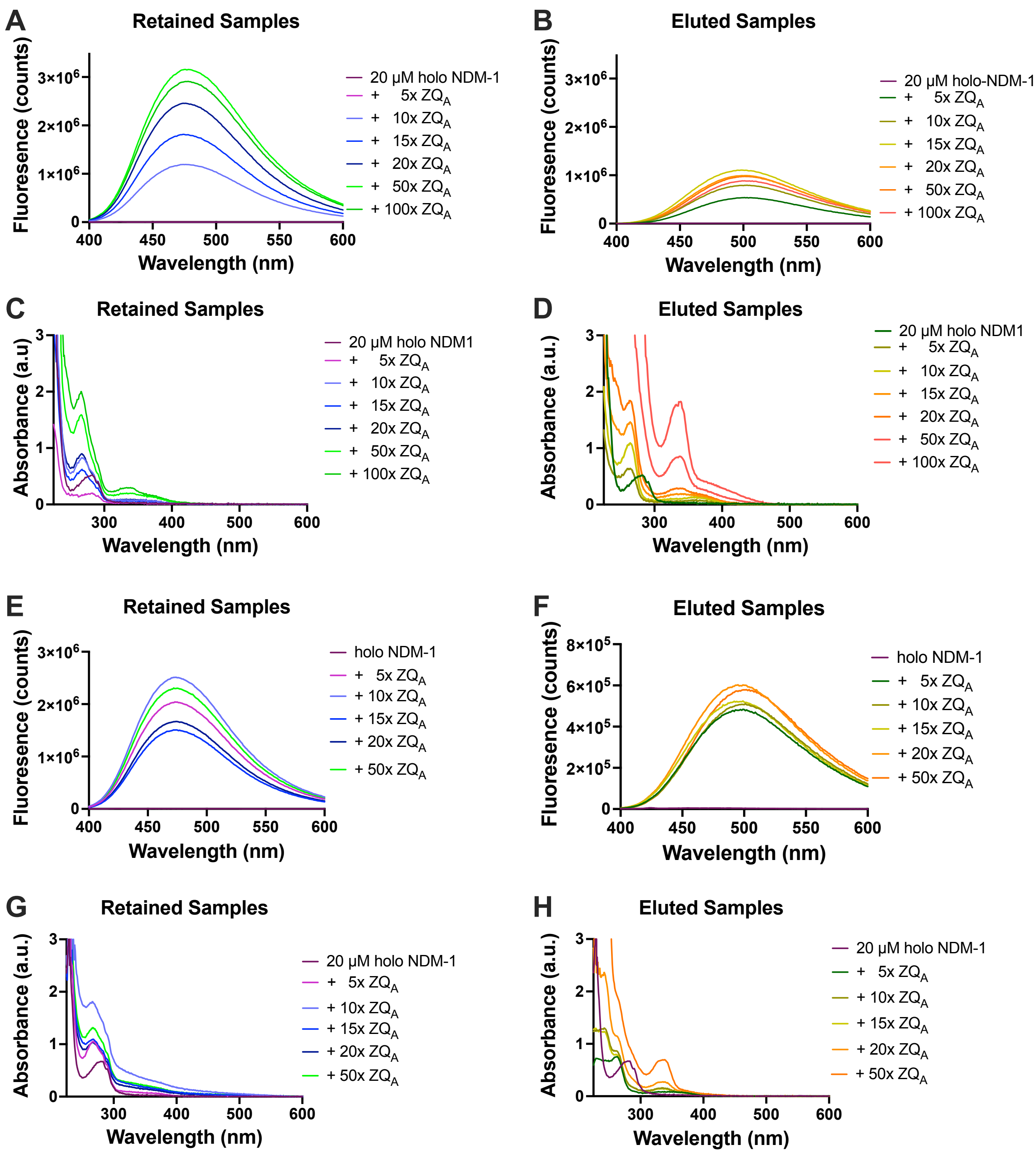

Figure S3. **Additional fluorescence and UV-vis spectra from fractionation studies.** Samples containing 20 µM holo NDM-1 in Storage Buffer (35 mM HEPES) were treated with 5–100 molar equivalents ZQ_A and fluorescence was analyzed for the (A, E) retained and (B, F) eluted samples along with UV-vis for the (C, G) retained and (D, H) eluted samples. (A, E) Retained fluorescence samples showed a blue shifted λ_{max} of 473 nm compared to the (B, F) eluted samples that had the expected λ_{max} of 500 nm for $[Zn(ZQ_A)_2]^{2-}$ complex in 35 mM HEPES (pH 7.5).

Quantitative HPLC/MS

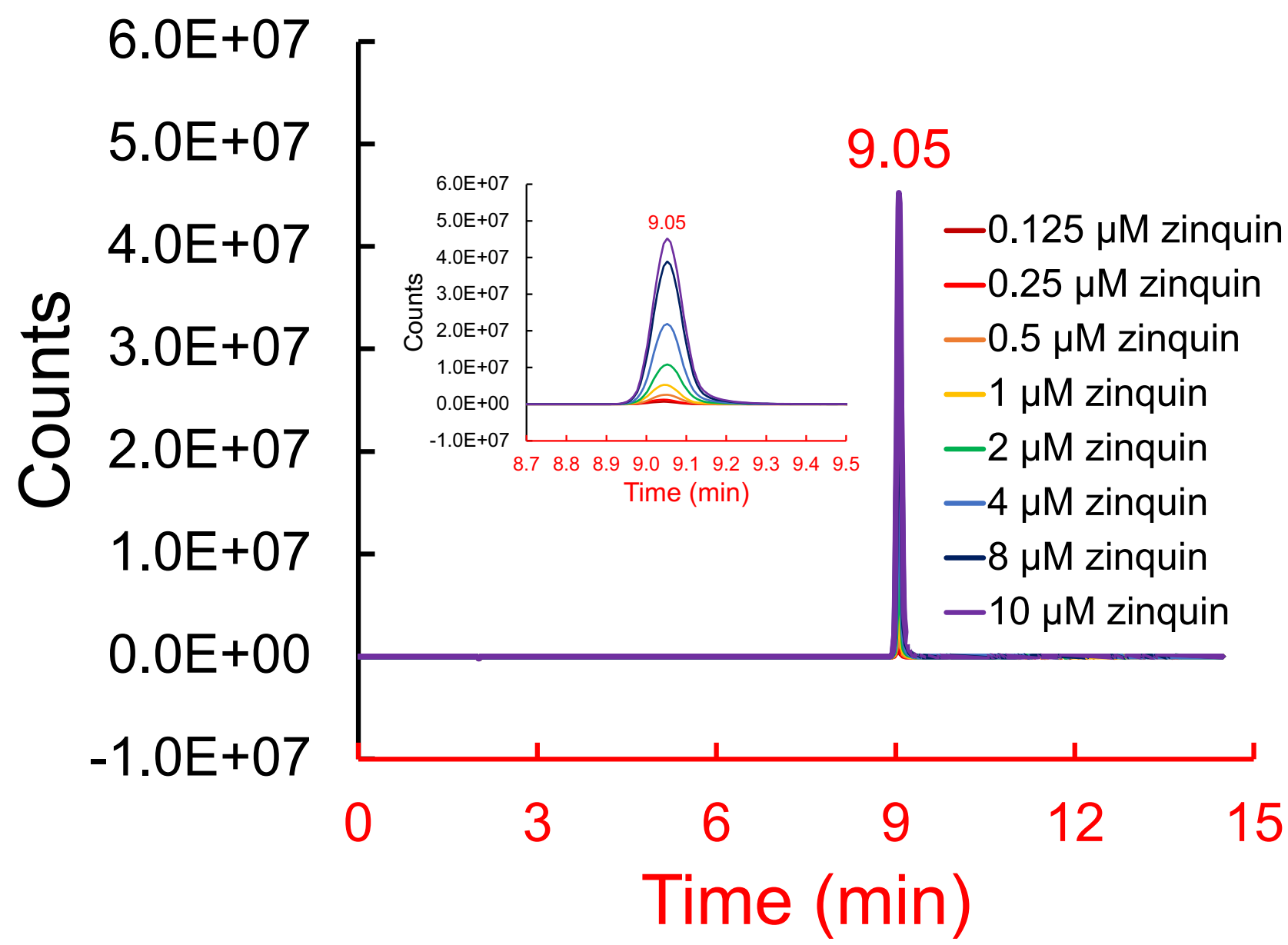

Figure S4. HPLC/MS extracted ion chromatograms (*m/z* = 381.7) of zinquin acid standard solutions.

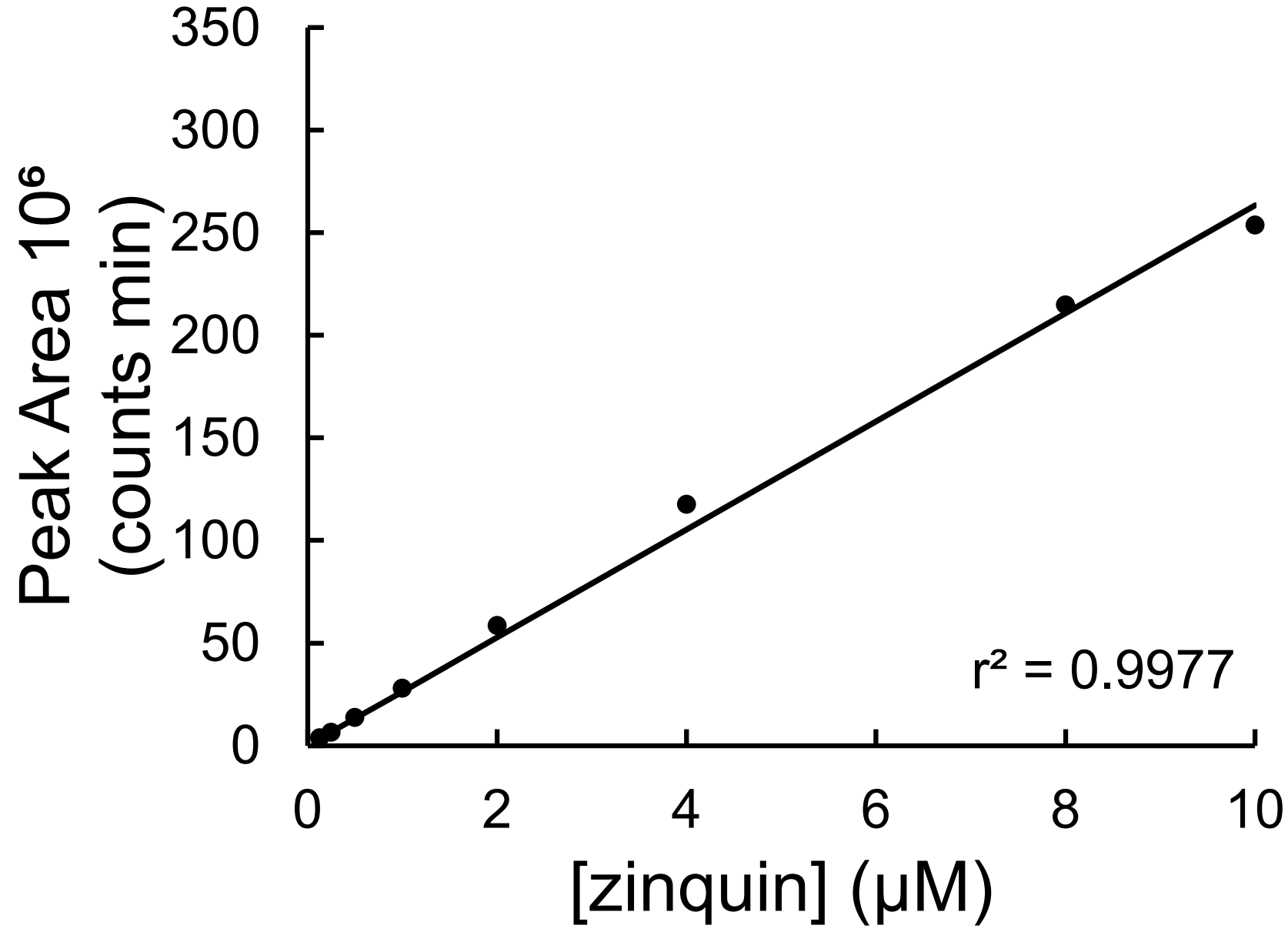

Figure S5. Standard curve from HPLC/MS integrated extracted ion chromatograms of zinquin acid standard solutions.

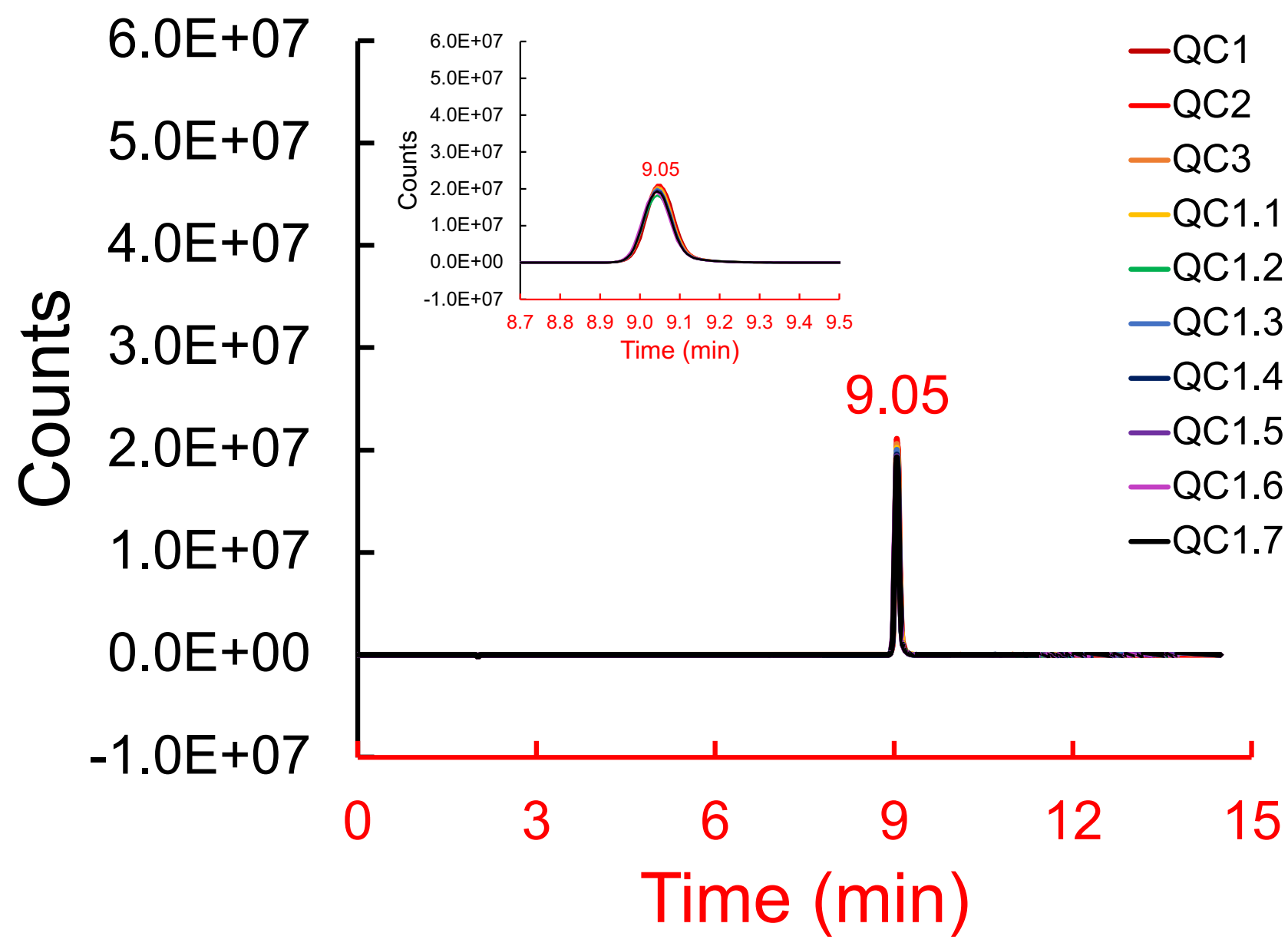

Figure S6. Zinquin acid quality control (QC) experiments. QC samples injected periodically, validating consistency of HPLC and MSD. QC 1, 2 and 3 traces are from independently prepared 4 µM samples. QC 1.X traces are replicate injections of QC sample 1.

XAS Fitting

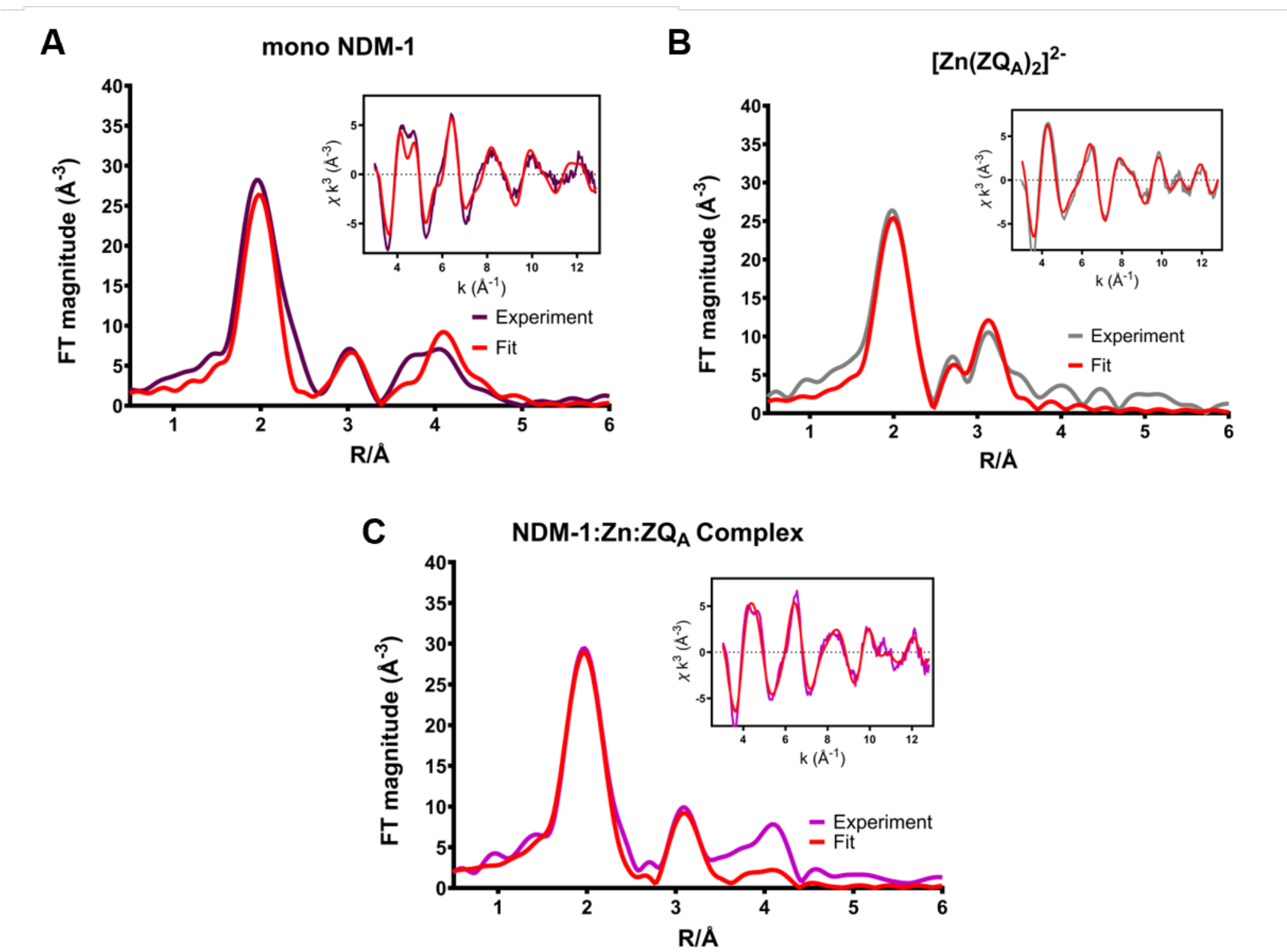

Figure S7. Zn EXAFS Fits and Fourier transforms for NDM-1, $[Zn(ZQ_A)]^{2-}$ complex, and ternary complex. Fit for the EXAFS data is shown in red for (A) 0.7 mM NDM-1 treated with 0.9 molar equivalents of $ZnSO_4$ (dark purple spectrum), (B) 2 mM ZQ_A treated with 0.9 molar equivalents $ZnSO_4$ (gray spectrum), and (C) complex formed in the retained portion of a spin filter between 0.7 mM NDM-1 bound to 0.9 molar equivalents $ZnSO_4$ and treated with 5 molar equivalents ZQ_A in Storage Buffer.

Table S1. Zn K-edge EXAFS Fitting Parameters.

Scattering Shell	N	R (Å)	DW ($Å^2$)	Fit Index	E_0 (ev)
**NDM-1 + Zn^{2+}**					
Zn-N (His)	4	2.013	0.009		
				0.74	-10.84
**2 ZQ_A + Zn^{2+}**					
Zn-N/O	2	1.978	0.001		
Zn-N/O	2	2.102	0.001		
Zn-O/N	2	3.123	0.001		
Zn-S	2	3.142	0.001		
Zn-C	4	2.884	0.013		
				0.58	-10.83
**holo NDM-1 + 5x ZQ_A Retained**					
Cu-N (His)	1	1.192	0.006		
Cu-N/O	2	1.967	0.001		
Cu-N/O	2	2.081	0.001		
Cu-O/N	2	3.189	0.001		
Cu-S	2	3.189	0.007		
				0.50	-10.39

Fluorescence Decay with Radiative Rate

Radiative rate measurements can provide information regarding relaxation of an electron and the number of environments excited electrons inhabit. To prepare a sample for radiative rate, a sample containing 10 µM holo NDM-1 and 10 molar equivalents of ZQ_A in 37.5 mM HEPES was prepared in the same manner as fractionation sample preparation. Time-correlated single photon counting (TCSPC) measurements were performed on the same FLS920 instrument with µF920H (Edinburgh Instruments) for excitation. Kinetic traces were fitted by Dr. Jiaqi Zhu using Surface Xplorer (Ultrafast System).

The 1-exponential spectral fit did not fit the exponential decay of the experimental data as well. However, for the 2-exponential fit, the t_1 value is likely an artifact of the IRF and not a real lifetime of the system given it is so close to the t_{IRF}. These fits would support that there is likely only one environment that the electron being excited is in, thus supporting a ternary complex centered around a Zn in the active site.

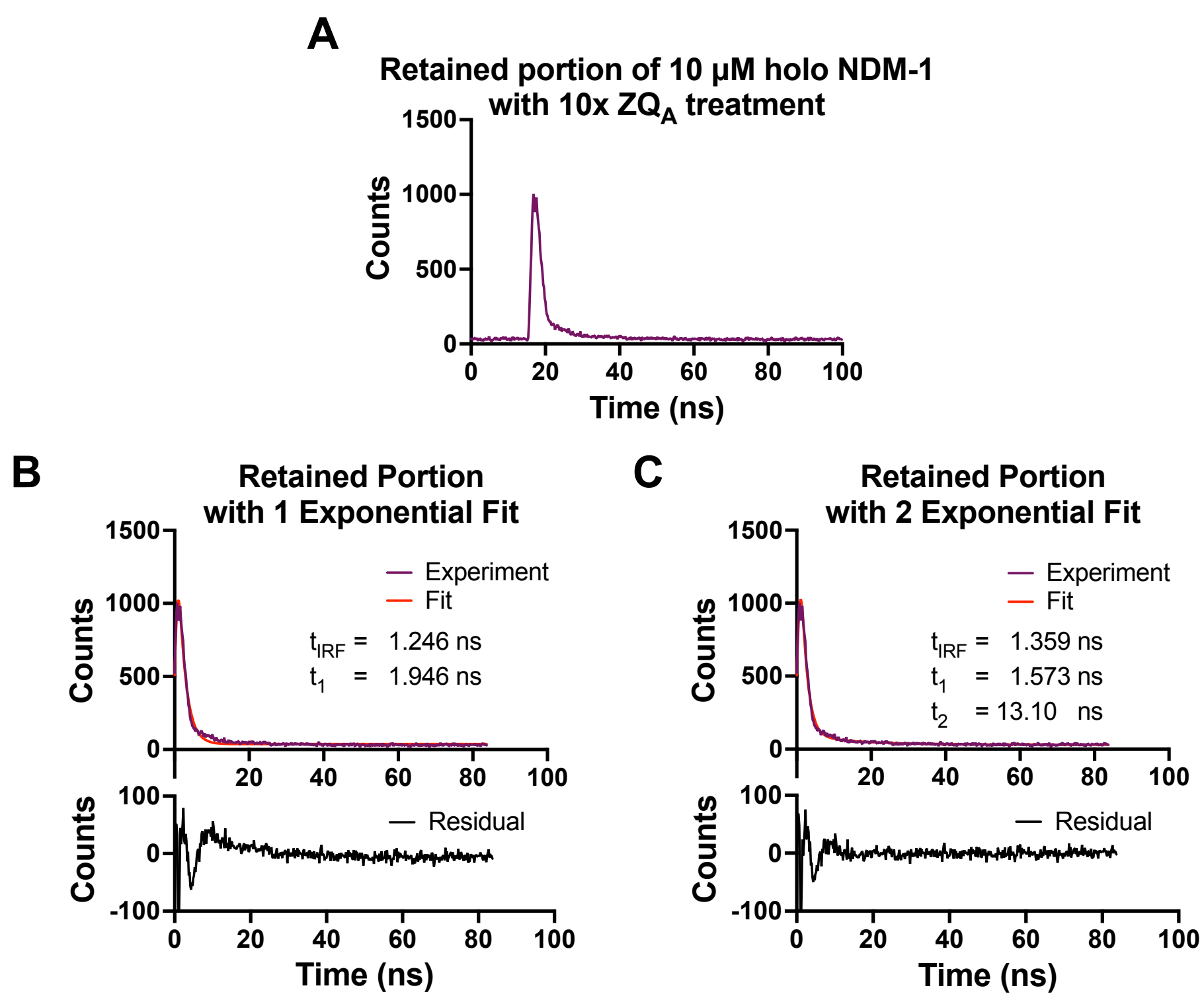

Figure S8. Radiative rate measurements for retained portion of 10 µM holo NDM-1 treated with 10 molar equivalents of ZQ_A. (A) Fluorescence decay spectra measured over a time frame of 100 ns. Spectrum was fit to (B) one exponential and (C) two exponentials.

Recipes for M9 Salts and Nutrients and M9 Media

The following solutions were made for preparation of M9 media.

- 10x M9 Salt Solution - 420 mM (60 g/L) Na_2HPO_4, 200 mM (30 g/L) KH_2PO_4, 85.5 mM (5 g/L) NaCl, pH 7.5
- 1000x (2 M) $MgSO_4$
- 1000x Trace Elements Mix - 50 mM HCl, 50 mM $FeCl_3$, 20 mM $CaCl_2$, 10 mM $MnCl_2$, 10 mM $ZnSO_4$, 2 mM $CuCl_2$, 2 mM $CoCl_2$, 2 mM $NiCl_2$
- 1000x (2 mM) Na_2MoO_4
- 1000x (2 mM) Na_2SeO_3
- 1000x (2 mM) H_3BO_3
- 100x Basal Medium Eagle Vitamin Solution - 200 mg/L i-inositol, 100 mg/L biotin, 100 mg/L choline chloride, 100 mg/L calcium D-pantothenate, 100 mg/L folic acid, 100 mg/L nicotinamide, 100 mg/L pyridoxal hydrochloride, 100 mg/L thiamine hydrochloride, 10 mg/L riboflavin, pH 9
- 10x (367 mM) ^{15}N-ammonium chloride, sterile filtered
- 50x (1.11 M, 200 g/L) glucose, sterile filtered

1760 mL of M9 Salts and Nutrients were prepared by diluting the following, in order, into 1530 mL of rapidly stirring DI-water; 1760 mL is the salt and nutrient portion of 2000 mL of this M9 media without 200 mL of 10x ^{15}N-ammonium chloride and 40 mL of 50x glucose, to be added later. The M9 Salts and Nutrients was filtered through a 0.22 µm sterile filter into an autoclaved glass bottle.

1. 2 mL of 1000x H_3BO_3
2. 2 mL of 1000x Na_2SeO_3
3. 2 mL of 1000x Na_2MoO_4
4. 2 mL of 1000x Trace Elements Mix
5. 2 mL of 1000x $MgSO_4$
6. 20 mL of 100x Basal Medium Eagle Vitamin Solution
7. 200 mL of 10x M9 Salt Solution

Characterization of Recombinant NDM-1

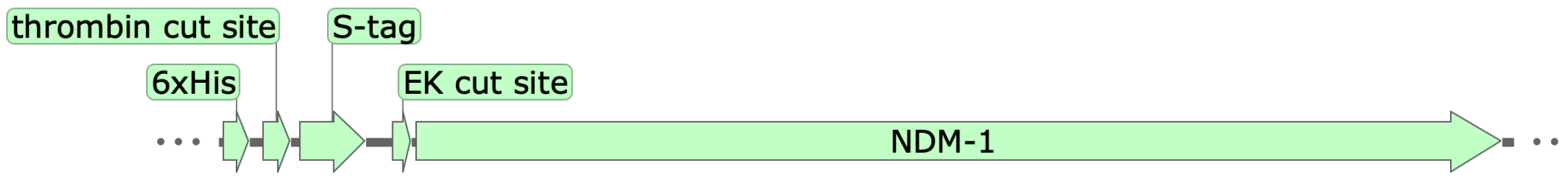

Figure S9. Illustration of open reading frame for expressed NDM-1 fusion protein from NDM-1_pET-30a(+) plasmid. The mature, translocated version of NDM-1 from *Escherichia coli*, lacking its physiological N-terminal signal sequence that is cleaved following periplasmic translocation, was expressed as a fusion protein with a 6xHis tag, thrombin cut site, S-tag, and Enterokinase (EK) cut site on the N-terminus. Fusion protein design enables isolation of 6xHis_NDM-1 fusion protein by IMAC, removal of N-terminal tag by 6xHis_EK, and purification of mature, recombinant NDM-1 by subtractive IMAC. A full, digital plasmid map is available for download from the Duke Research Data Repository: https://doi.org/10.7924/r4xg9wb2w.

Amino acid sequence of 6xHis_NDM-1 fusion protein:
MHHHHHHSSGLVPRGSGMKETAAAKFERQHMDSPDLGTDDDDKAMGEIRPTIGQQMETGDQ
RFGDLVFRQLAPNVWQHTSYLDMPGFGAVASNGLIVRDGGRVLVVDTAWTDDQTAQILNWIK
QEINLPVALAVVTHAHQDKMGGMDALHAAGIATYANALSNQLAPQEGMVAAQHSLTFAANGW
VEPATAPNFGPLKVFYPGPGHTSDNITVGIDGTDIAFGGCLIKDSKAKSLGNLGDADTEHYAAS
ARAFGAAFPKASMIVMSHSAPDSRAAITHTARMADKLR.

Deconvoluted HRMS (ESI): calculated for $[C_{1336}H_{2076}N_{390}O_{411}S_{13}]$: 30594.33; found: 30594.45.
Deconvoluted HRMS (ESI): calculated for $[C_{1336}H_{2076}{}^{15}N_{390}O_{411}S_{13}]$: 30981.49; found: 30963.77; 95 % ^{15}N labeled

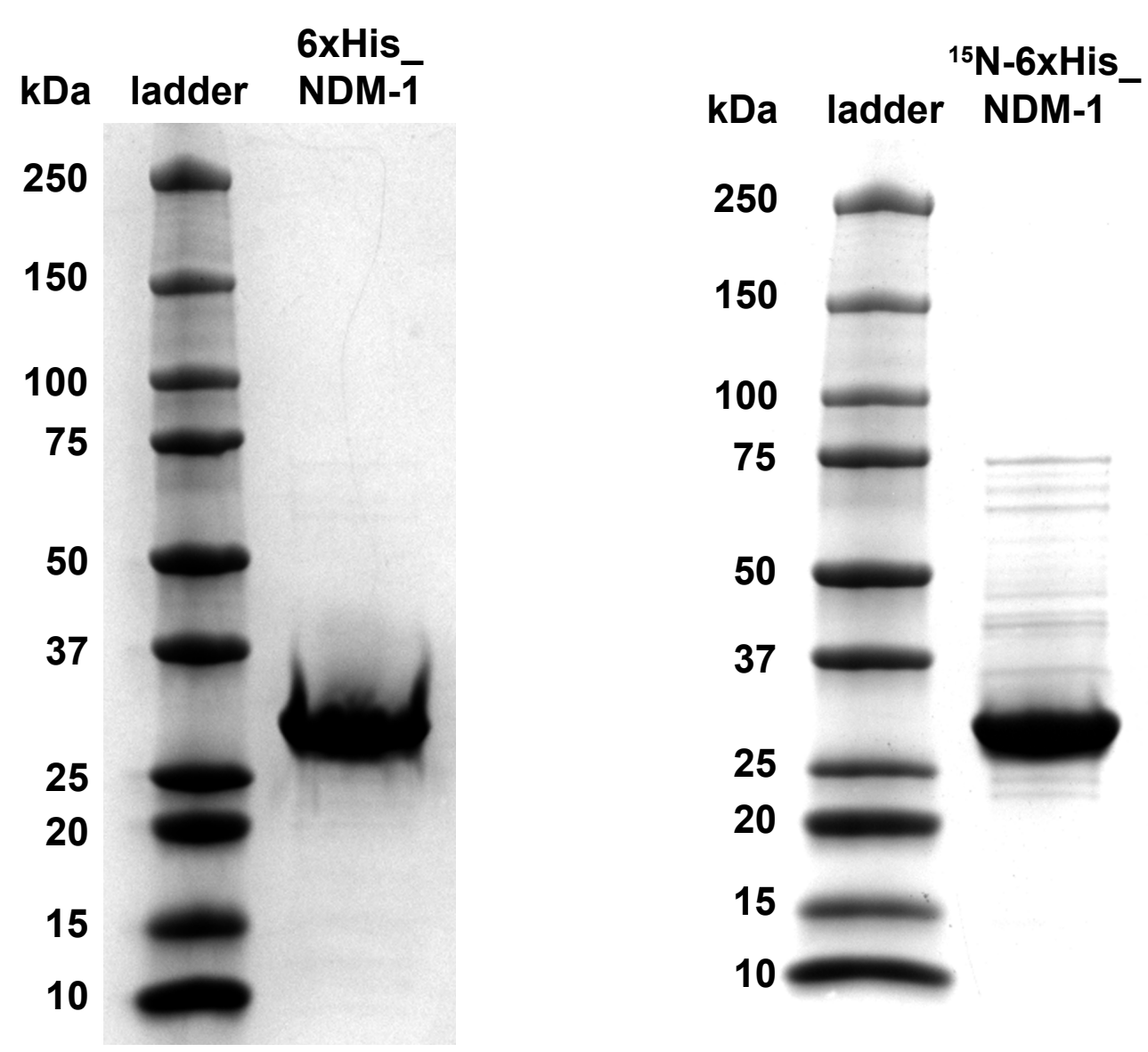

Figure S10. Denatured gel electrophoresis of 6xHis_NDM-1 and ^{15}N-6xHis_NDM-1. Sample as purified from FPLC.

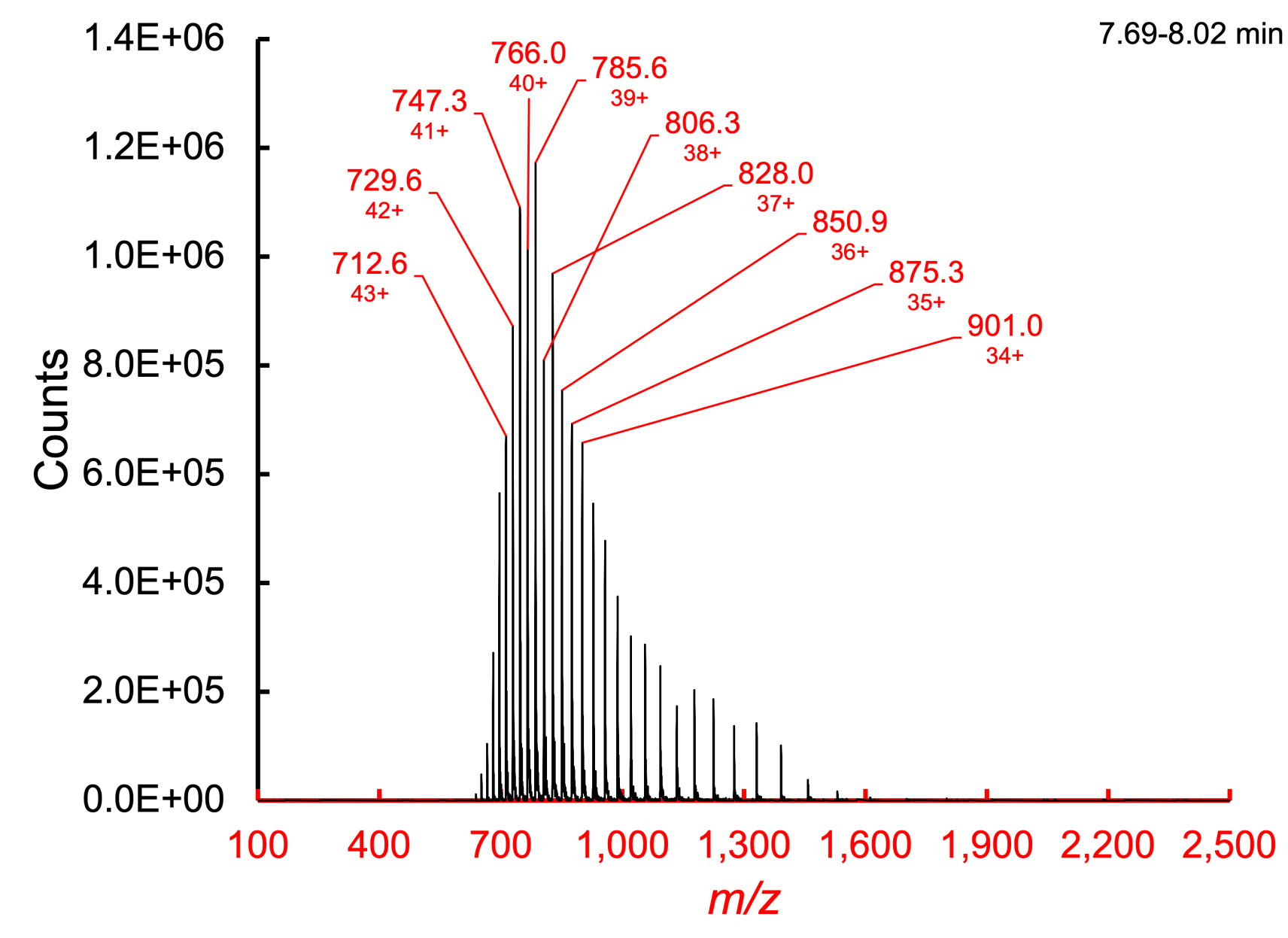

Figure S11. MS (ESI) of 6xHis_NDM-1. Select charge states indicated. Sample as purified from FPLC.

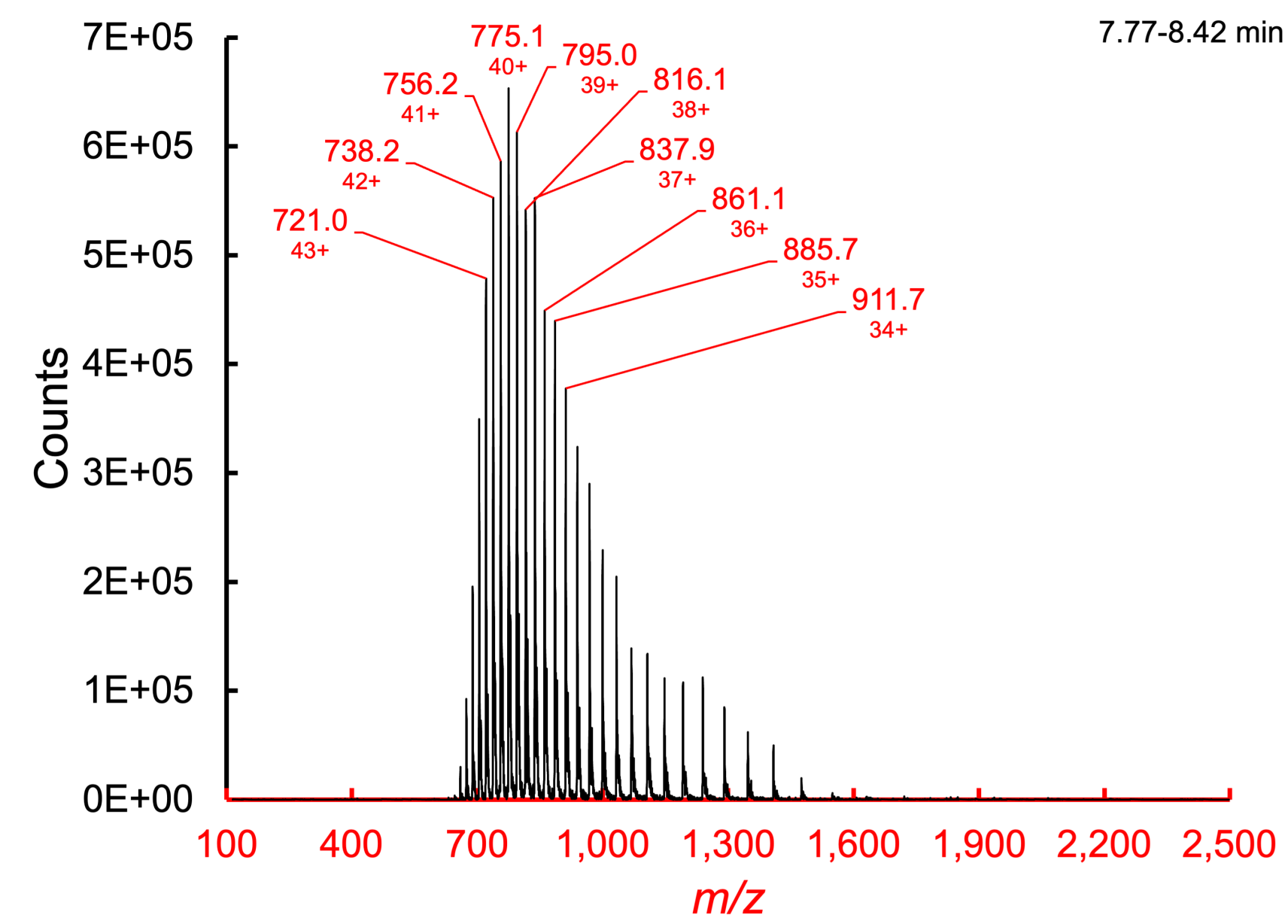

Figure S12. MS (ESI) of ^{15}N-6xHis_NDM-1. Select charge states indicated. Sample as purified from FPLC.

Amino acid sequence of recombinant NDM-1:
AMGEIRPTIGQQMETGDQRFGDLVFRQLAPNVWQHTSYLDMPGFGAVASNGLIVRDGGRVLV
VDTAWTDDQTAQILNWIKQEINLPVALAVVTHAHQDKMGGMDALHAAGIATYANALSNQLAPQ
EGMVAAQHSLTFAANGWVEPATAPNFGPLKVFYPGPGHTSDNITVGIDGTDIAFGGCLIKDSKA
KSLGNLGDADTEHYAASARAFGAAFPKASMIVMSHSAPDSRAAITHTARMADKLR.

Deconvoluted HRMS (ESI): calculated for $[C_{1137}H_{1773}N_{323}O_{345}S_{10}]$: 25808.14; found: 25808.40.
Deconvoluted HRMS (ESI): calculated for $[C_{1137}H_{1773}{}^{15}N_{323}O_{345}S_{10}]$: 26128.78; found: 26115.21; 96 % ^{15}N labeled

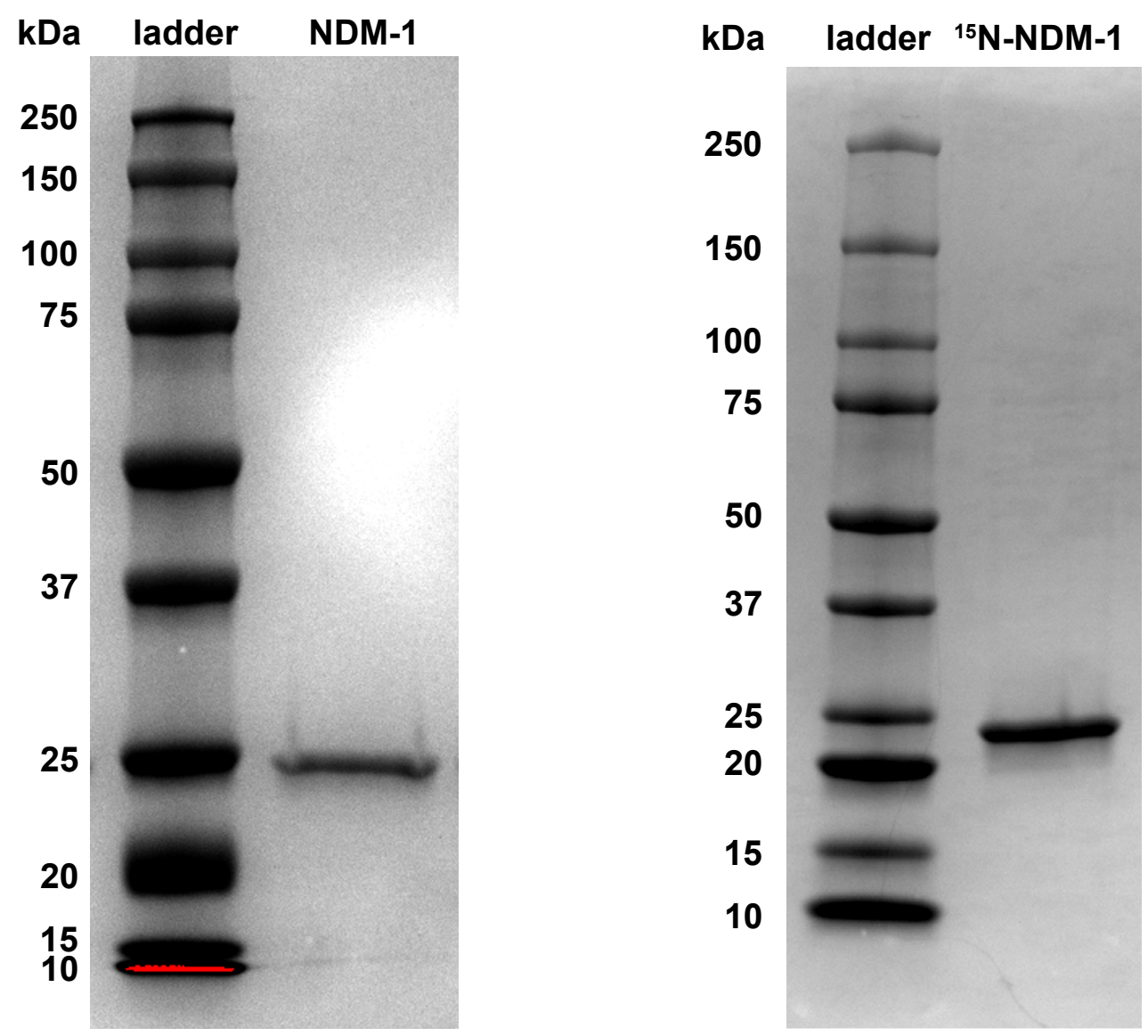

Figure S13. Denatured gel electrophoresis of recombinant NDM-1. Sample as purified from FPLC.

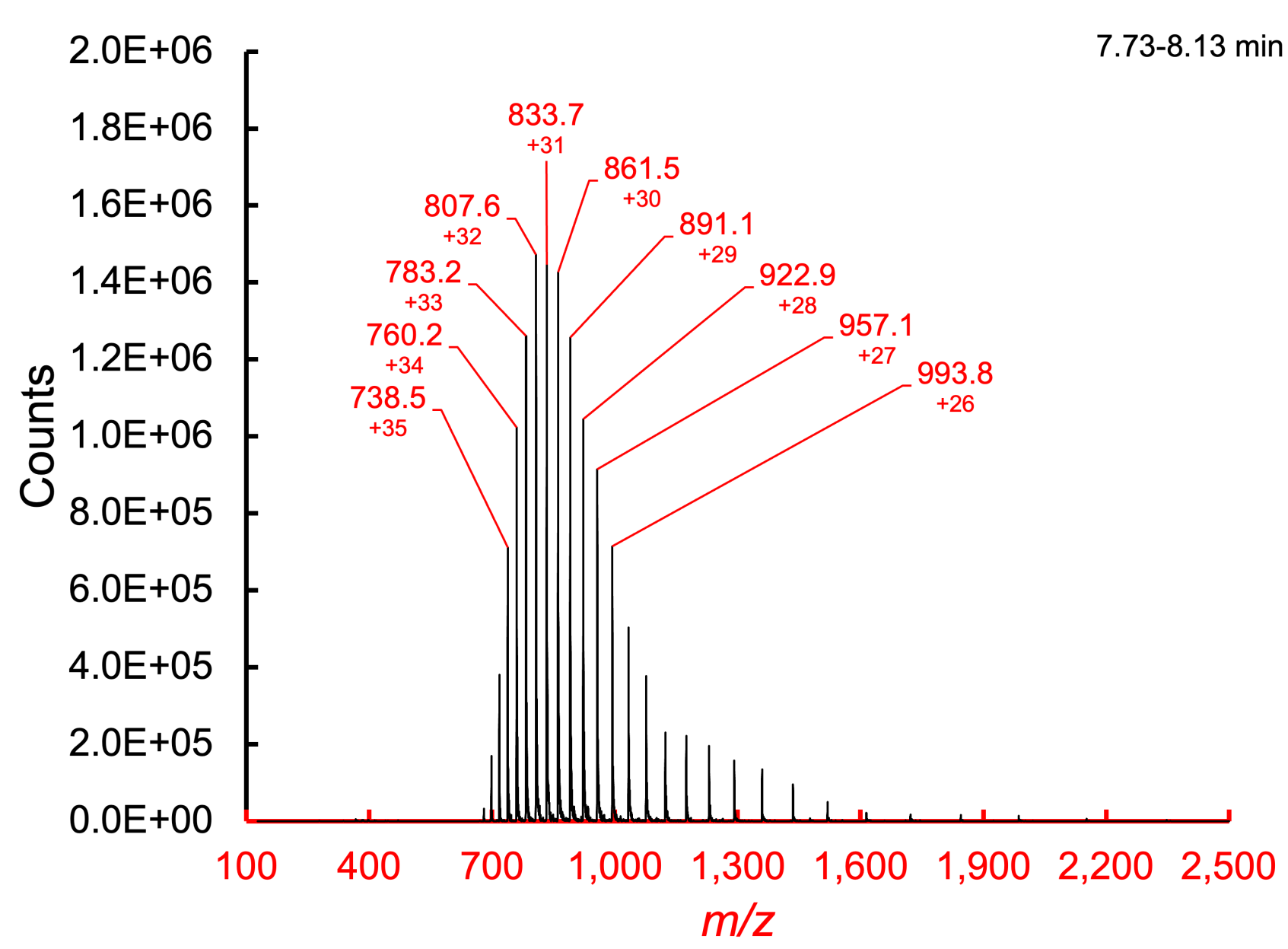

Figure S14. MS (ESI) of recombinant NDM-1. Select charge states indicated. Sample as purified from FPLC.

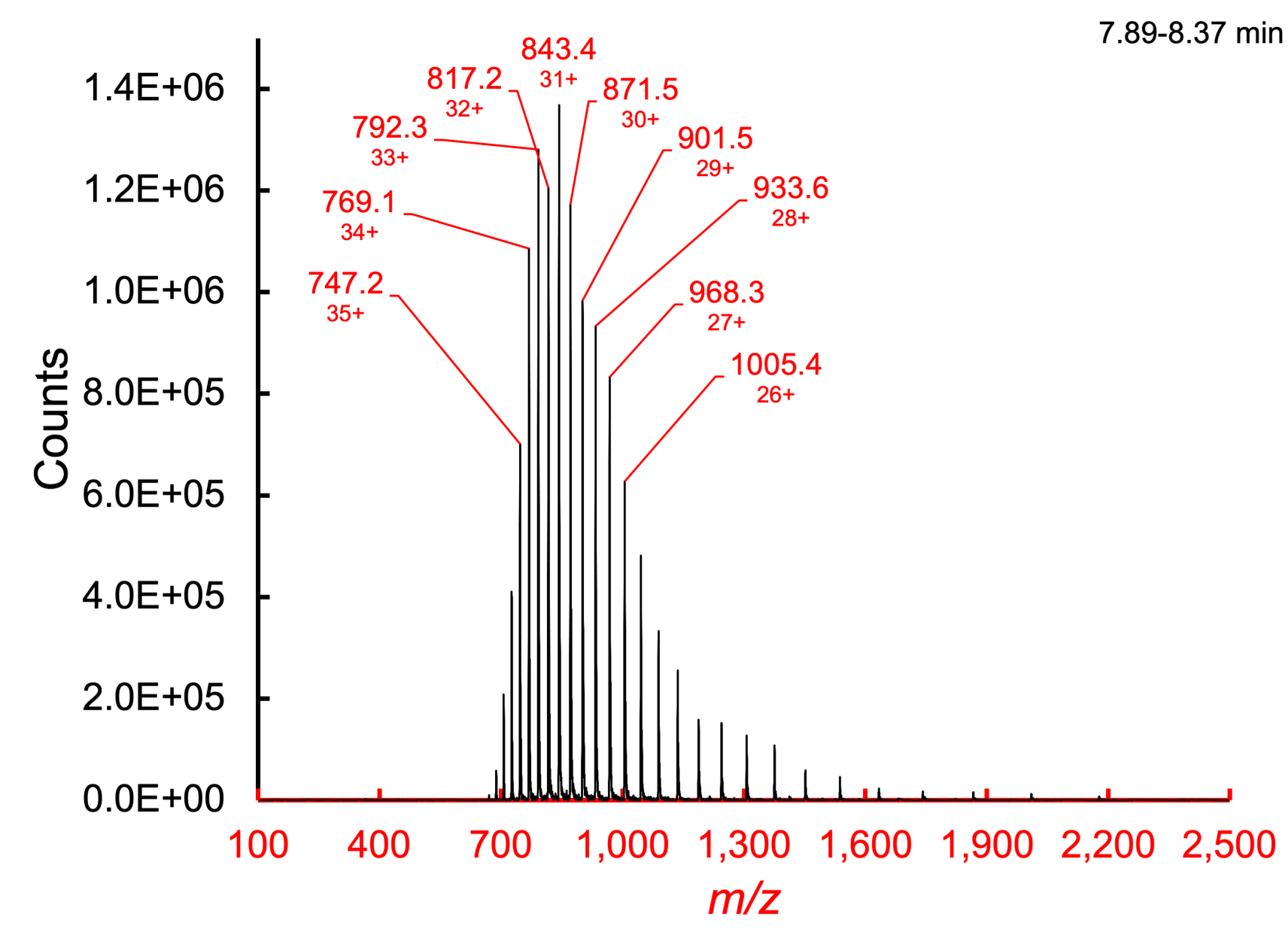

Figure S15. MS (ESI) of recombinant ^{15}N-NDM-1. Select charge states indicated. Sample as purified from FPLC.

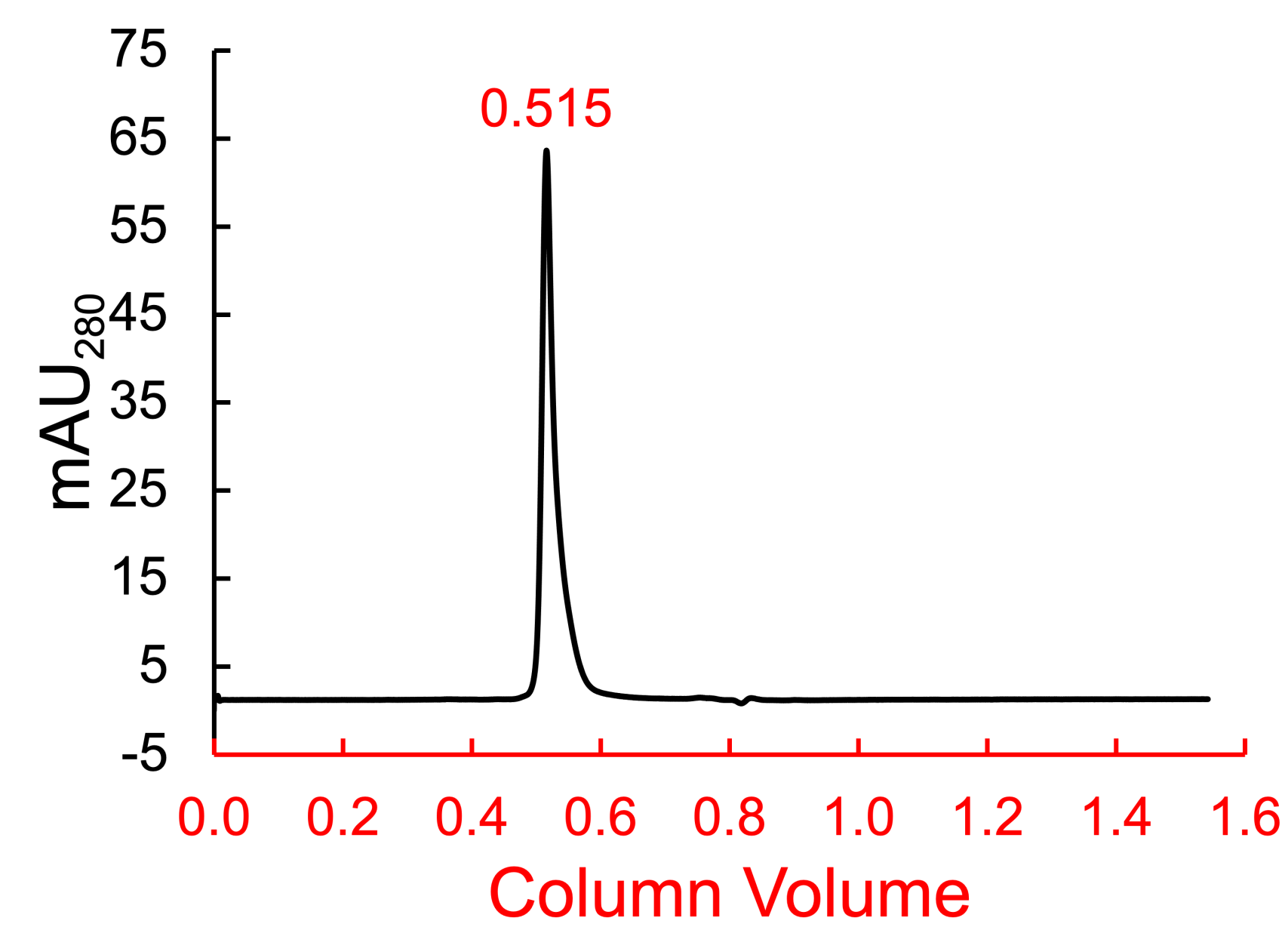

Figure S16. SEC of recombinant NDM-1. Sample as purified from FPLC.

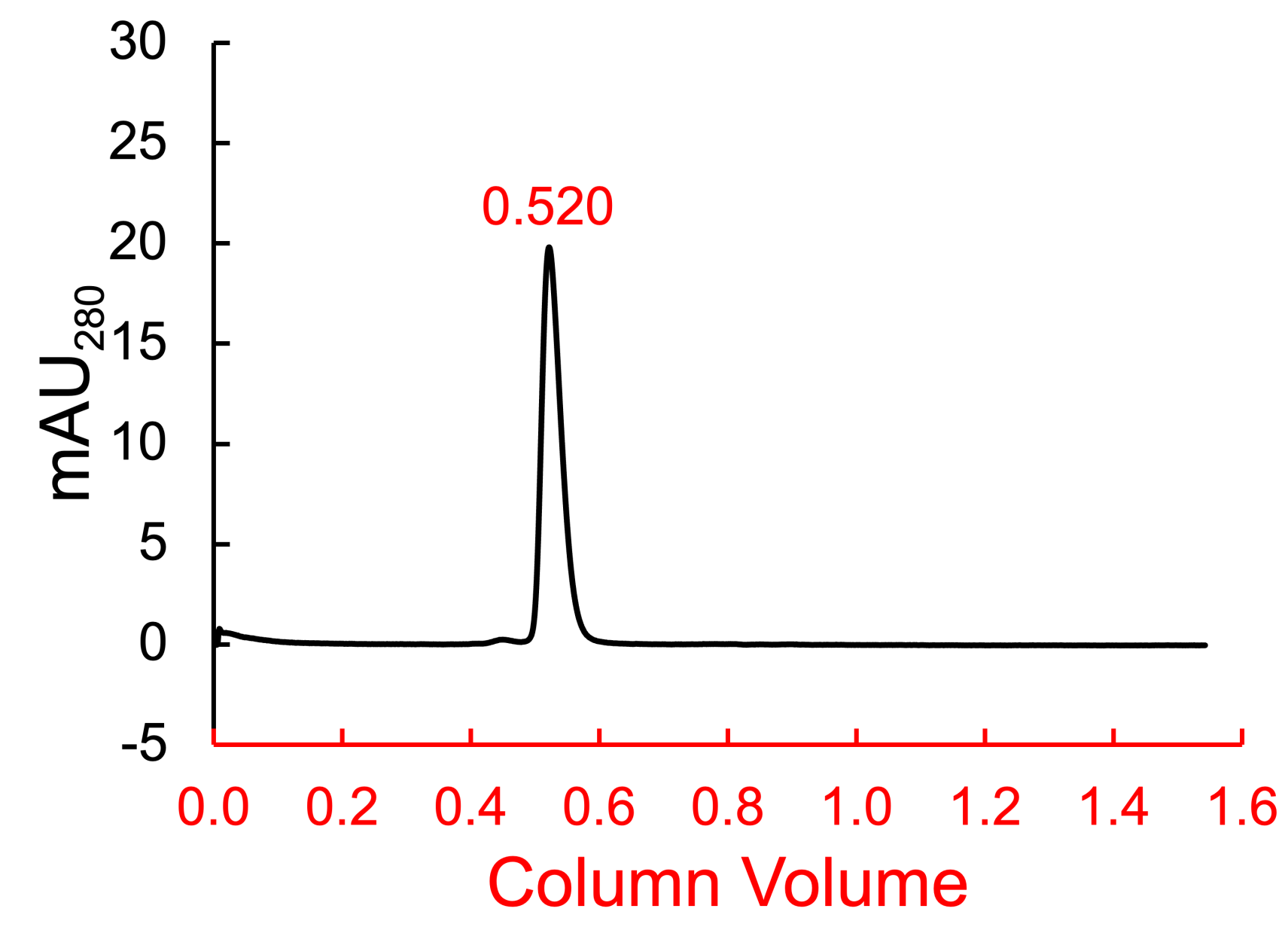

Figure S17. SEC of recombinant ^{15}N-NDM-1. Sample as purified from FPLC**.**

^{15}N-^{1}H HSQC NMR Titrations

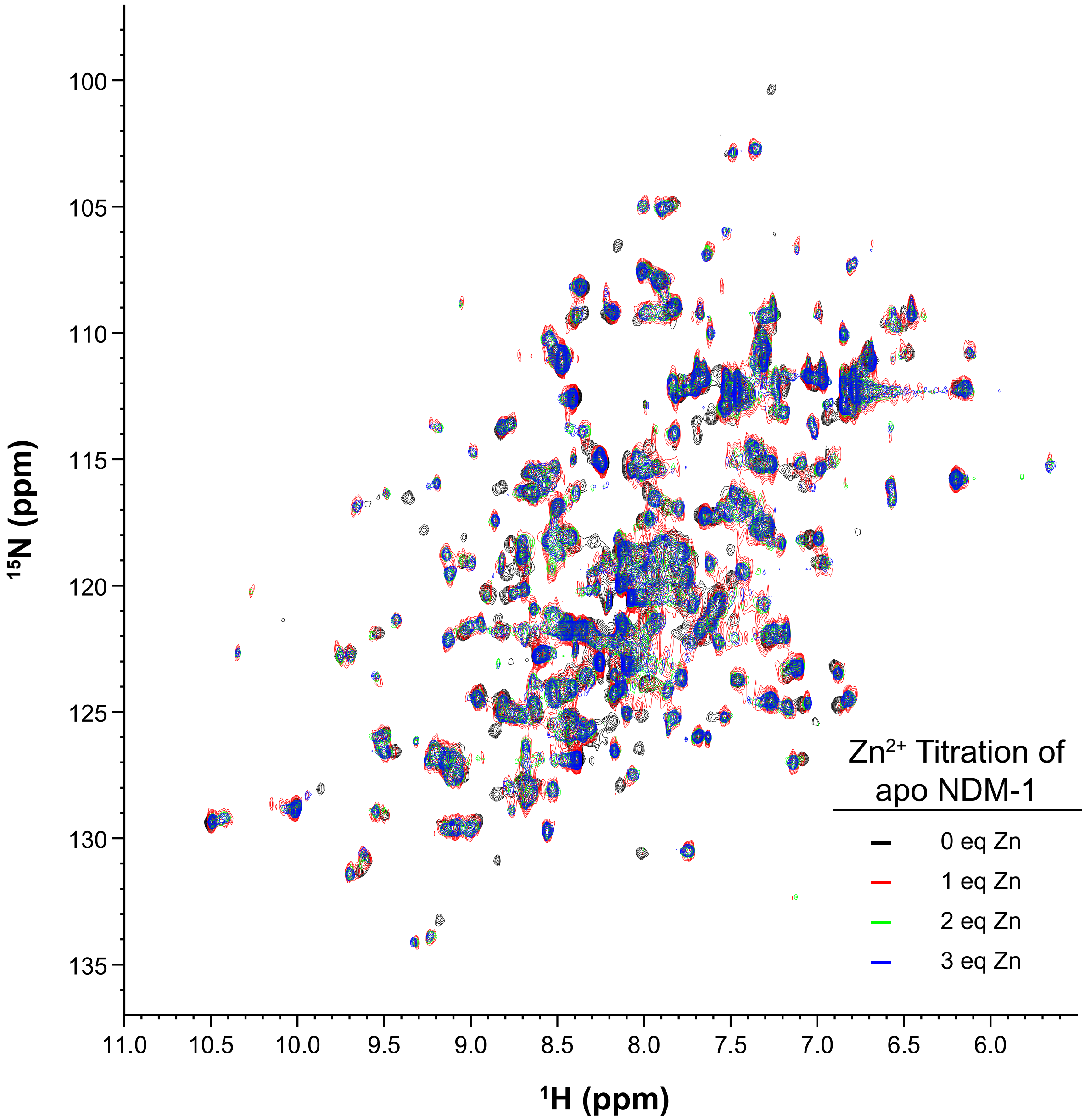

Figure S18. ^{15}N-^{1}H HSQC NMR of apo ^{15}N-NDM-1 titrated with $ZnSO_4$. Apo NDM-1 structure is distinct from NDM-1 with monoZn and holo NDM-1. Holo NDM-1's structure is more stable, but similar to mono Zn NDM-1.

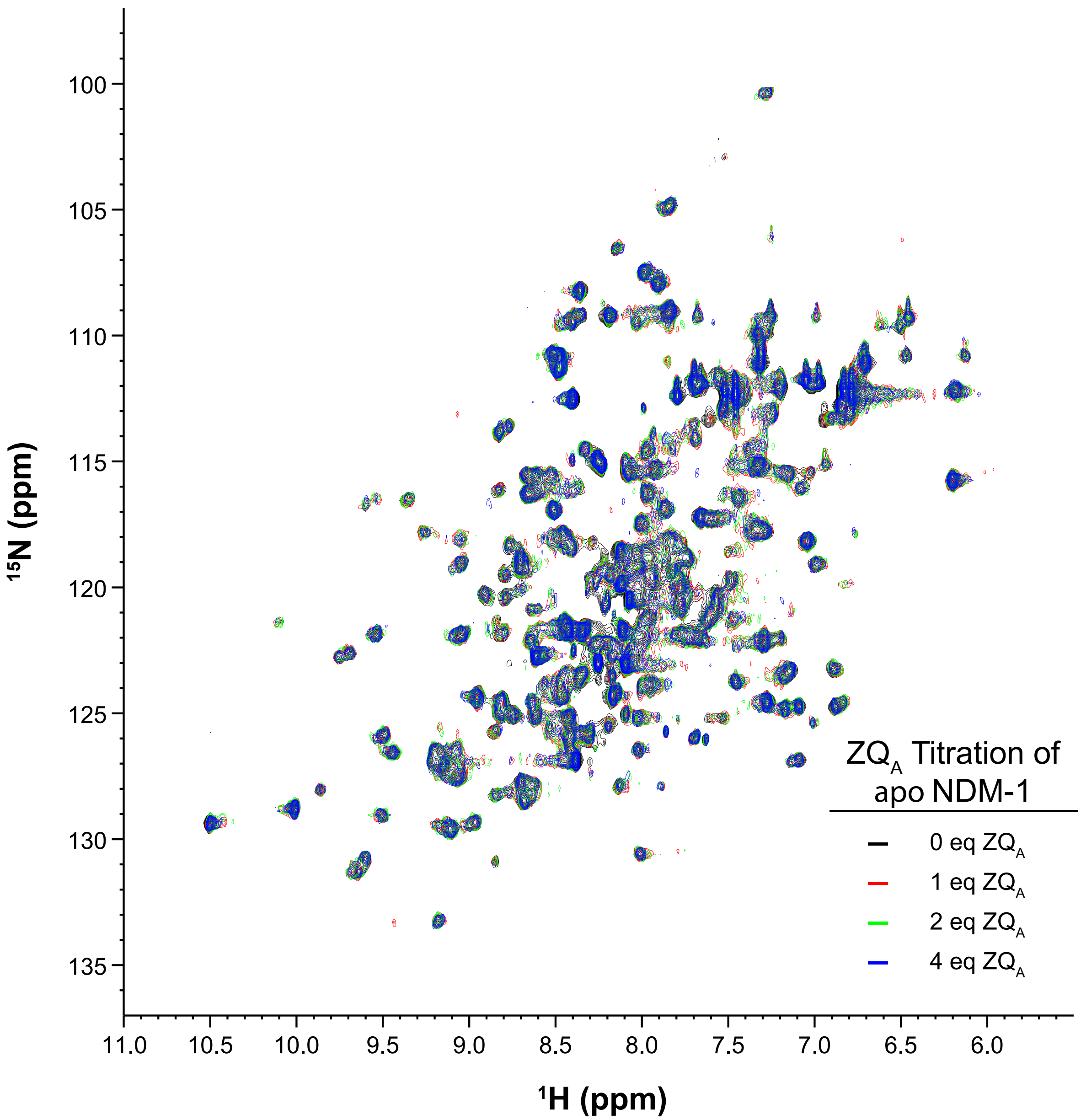

Figure S19. ^{15}N-^{1}H HSQC NMR of apo ^{15}N-NDM-1 titrated with ZQ_A. Apo NDM-1's structure does not change with addition of ZQ_A, indicating no association.

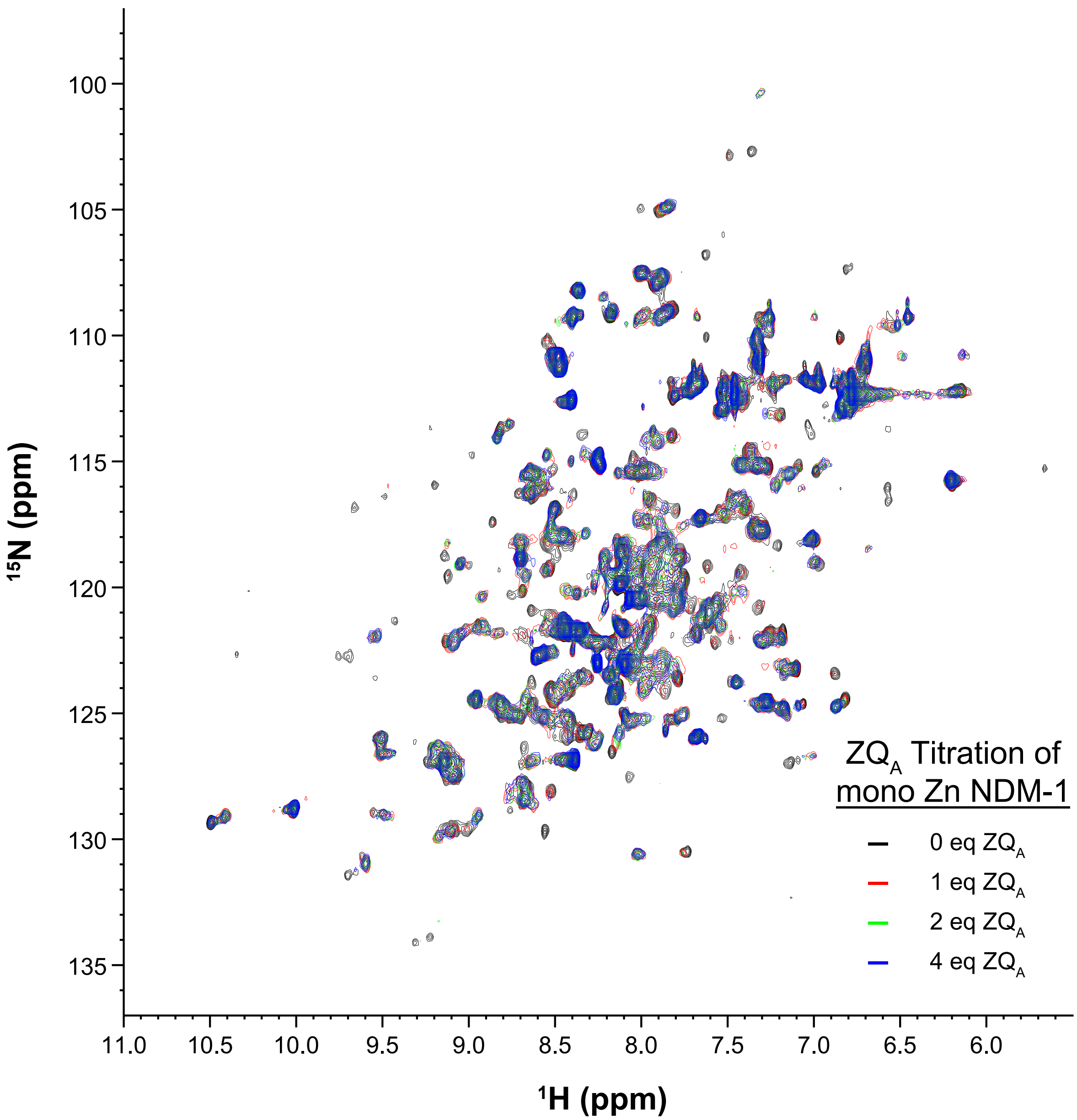

Figure S20. ^{15}N-^{1}H HSQC NMR of monoZn ^{15}N-NDM-1 titrated with ZQ$_A$. MonoZn NDM-1's structure changes with addition of ZQ$_A$, indicating a zinc-dependent small-molecule/protein association.

MD Simulations

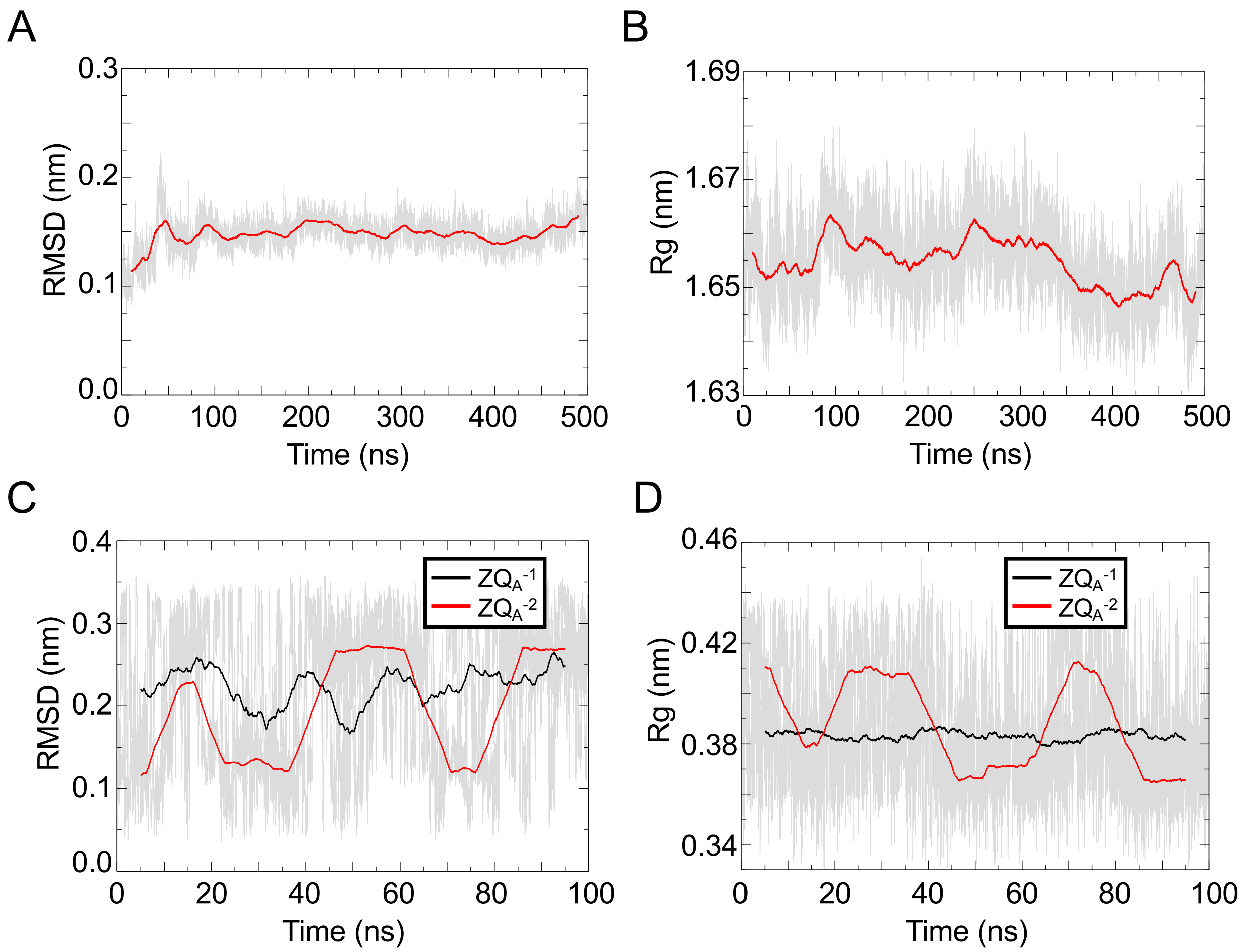

Figure S21. RMSD and Rg from MD Simulations. Plots for NDM-1 (A and B) and both charge states of ZQ_A (C and D). For clarity the raw data is shown in grey color, while a running average is shown in bold solid line. ZQ_A^{-1} and ZQ_A^{-2}, in panels C and D, are colored black and red, respectively.
